# Protocol for a pilot randomized controlled trial of a telehealth-delivered counseling intervention to reduce suicidality and improve HIV care engagement in Tanzania

**DOI:** 10.1371/journal.pone.0289119

**Published:** 2023-07-27

**Authors:** Brandon A. Knettel, Elizabeth T. Knippler, Ismail Amiri, Louise Joel, Kim Madundo, Elizabeth F. Msoka, Judith Boshe, Clotilda S. Tarimo, Victor Katiti, Jackline Rwakilomba, Elizabeth L. Turner, Linda Minja, Catherine A. Staton, Joao Ricardo N. Vissoci, Blandina T. Mmbaga, Michael V. Relf, David B. Goldston

**Affiliations:** 1 Duke University School of Nursing, Durham, NC, United States of America; 2 Duke Global Health Institute, Durham, NC, United States of America; 3 Duke Center for AIDS Research, Durham, NC, United States of America; 4 Kilimanjaro Christian Medical Centre, Moshi, Tanzania; 5 Kilimanjaro Clinical Research Institute, Moshi, Tanzania; 6 Kilimanjaro Christian Medical University, Moshi, Tanzania; 7 Duke Department of Biostatistics & Bioinformatics, Durham, NC, United States of America; 8 Duke Department of Emergency Medicine, Durham, NC, United States of America; 9 Department of Psychiatry & Behavioral Sciences, Duke University, Durham, NC, United States of America; PLoS ONE, UNITED STATES

## Abstract

**Objective:**

Suicidal ideation is strikingly common among people living with HIV (PLWH) worldwide, leading to higher burden of disease, poor HIV care engagement, and loss of life. In low- and middle-income countries such as Tanzania, mental health resources are scarce, requiring innovative strategies for treatment. We describe the protocol for a clinical trial of a three-session telehealth counseling intervention to reduce suicidality and improve HIV care engagement in Tanzania.

**Methods:**

In a pilot randomized controlled trial, we will assess the feasibility, acceptability, and potential efficacy of a new telehealth intervention, termed “IDEAS for Hope”. A total of 60 PLWH will be enrolled from two HIV clinics in the Kilimanjaro region and connected to telehealth counsellors based at a large regional hospital. Participants will be ≥18 years old and speak either Kiswahili or English. Patient screening will occur during routine HIV clinical care to identify PLWH experiencing suicidal ideation. Baseline surveys will be administered upon enrollment and participants will be randomized 1:1 to receive either IDEAS for Hope or the comparison condition, a brief safety planning session. All participants will receive an additional referral for psychiatric treatment. Follow-up assessment will occur at three months. IDEAS for Hope is informed by a Motivational Interviewing-enhanced safety planning intervention (MI-SafeCope) and our formative work in Tanzania. The model consists of Four Pillars: living healthy with HIV, managing HIV stigma, seeking social support, and meeting basic needs. Together, these mechanisms serve as a foundation for developing a sense of safety and hope for the future. Outcome measures will include intervention feasibility, acceptability, participant suicidality, and HIV care engagement.

**Significance:**

Innovative, telehealth-based counseling represents a promising treatment for suicidality among PLWH in low-resource settings. Results from this pilot trial will inform intervention refinement and parameter estimates for a future clinical trial powered to evaluate effectiveness.

## Introduction

Tanzania has an estimated population of 1.7–1.9 million people living with HIV (PLWH), or approximately 4.5% of the adult population [1]. Depression and suicidal ideation disproportionately impact PLWH, with rates drastically exceeding that of the general public [2, 3]. The World Health Organization estimates over 3000 people die by suicide in Tanzania each year, and studies have indicated that approximately 26% of deaths by suicide in Tanzania are among PLWH [4, 5]. Routine screening for suicidality in HIV care, however, has not been implemented.

Studies within the Kilimanjaro region of Tanzania have shown that suicidal ideation among PLWH is often driven by misinformation and stigma related to HIV, including beliefs and worries about one’s prognosis, transmission risk, social standing, and future life [2, 5]. Misinformation and stigma can also contribute to internalized feelings of shame, enacted mistreatment from others, social isolation, and diminished financial opportunities, which exacerbate mental health challenges [6, 7]. The impacts of these factors on HIV outcomes are devastating, as mental health challenges among PLWH are associated with delayed HIV treatment, poor HIV care engagement, increased transmission risk, and reduced life expectancy [8–12].

Despite these myriad challenges, when PLWH have access to evidence-based mental health treatment that incorporates sound adherence counseling, HIV care engagement and treatment outcomes improve [13–15]. Integrating counseling services into HIV clinical care in low-income countries such as Tanzania, however, is challenging. In our recent scoping review of counseling for suicide prevention in Africa, we identified only 11 intervention studies ever conducted on the topic, including only three that focused on supporting PLWH [16]. Other intervention models have been developed in high-income countries but are resource-intensive, reducing their feasibility for implementation in low-resource settings [3, 17]. Indeed, Tanzania is among the world’s most under-served and overburdened nations for mental health care, with just 55 psychologists and psychiatrists providing care coverage for over 60 million people [18]. Novel, cost-effective, low-burden approaches are thus needed to address the unique needs of PLWH experiencing suicidality where mental health specialists are rare.

Telehealth approaches for mental health care provide a promising opportunity for ‘leapfrogging,’ whereby low-income countries can bypass unsuccessful or unfeasible strategies to reach or surpass the benefits of treatments available in high-income countries [19]. The Tanzanian health system uses a ‘hub and spoke’ model whereby smaller health clinics and hospitals refer high-acuity cases to specialists at larger medical centers. Telehealth approaches, however, can facilitate immediate remote referral, reducing the burden on both patients and healthcare professionals [20]. Combined with the rapid growth in mobile technology, smartphones, and internet access in Tanzania, telehealth care may facilitate treatment access for PLWH who are experiencing suicidal ideation and reduce the mental health treatment gap.

Task-sharing is another critical component of building mental health treatment capacity in low-resource settings. Multiple global studies have demonstrated the potential for health care workers other than psychologists and psychiatrists to effectively provide assessment and counseling under the supervision of mental health specialists [21, 22]. This may include community health workers, nurses, and allied health professionals [23, 24]. HIV clinic providers often feel overwhelmed in their existing duties, leaving little bandwidth to offer additional supportive counseling [25]. In this context, there is great potential to shift the few mental health providers to a supervisory role focused on training and overseeing the work of non-specialists, which can contribute to rapid increases in treatment capacity.

This pilot study aims to test the feasibility, applicability, and potential efficacy of the first clinic-based telehealth counselling intervention developed to reduce suicidality and improve HIV care engagement in Tanzania. The intervention will employ task-sharing by training non-mental health specialists, Diploma-level nurses (equivalent to a Licensed Practical Nurse), to provide telehealth counseling. The intervention model is designed for “addressing suicidal (I)deation and (D)epression through HIV (E)ducation and counseling, advancing treatment (A)dherence, and reducing (S)tigma for fostering (Hope) and resilience among people living with HIV” (IDEAS for Hope). The IDEAS for Hope framework consists of Four Pillars for building safety and hope for the future: 1) living healthy with HIV, 2) managing HIV stigma, 3) seeking the support of others, and 4) actively problem-solving to meet basic needs. These pillars are anchored to personal values, including the improved health that comes from the successful management of HIV. The intervention is informed by Motivational Interviewing-enhanced safety planning for suicide prevention (MI-SafeCope) [26], and draws from prior work identifying appropriate intervention characteristics for PLWH experiencing suicidality and other mental health challenges [9, 14, 27, 28]. Translated study tools have been validated for use in the Tanzania region.

## Materials and methods

### Study design

This IDEAS for Hope pilot randomized control trial (clinicaltrials.gov identifier: NCT 04696861) aims to assess the feasibility, acceptability, and potential efficacy of a three-session, telehealth-based counseling intervention designed to reduce suicidality and enhance HIV care engagement among PLWH in Kilimanjaro, Tanzania. Patient recruitment is estimated to begin in May 2023 and conclude by May 2024. During routine HIV care appointments, clinic nurses will administer a 3-question screener that includes a single yes/no question to assess suicidal ideation, derived from the Columbia–Suicide Severity Rating Scale (C-SSRS) Screen Version [29]: “In the last month, have you had any actual thoughts of killing yourself?” and the 2-item Patient Health Questionnaire-2 (PHQ-2) to assess for depression [30].

**Fig 1:**
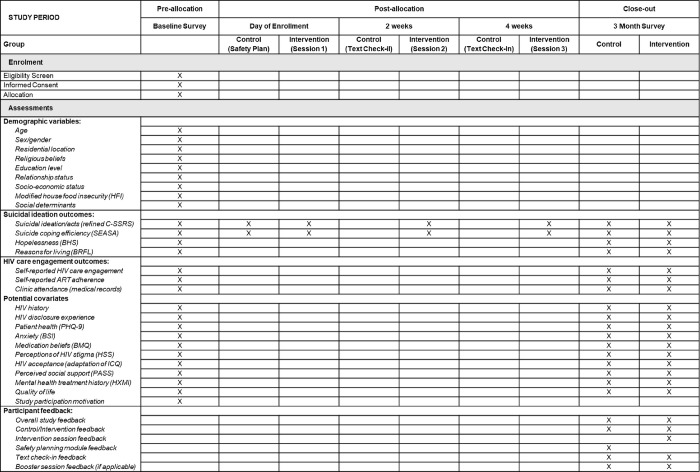
SPIRIT schedule of enrolment, interventions, assessments. ART: antiviral therapy, BHS: Beck Hopelessness Scale, BRFL: Brief Reasons for Living Inventory, BSI: brief symptom inventory, BMQ: Beliefs about Medicines Questionnaire, C-SSRS: Columbia-Suicide Severity Rating Scale, HSS: HIV Stigma Scale, HXMI: History of Mental Health Illness & Treatment, ICQ: Illness Cognition Questionnaire, PASS: Perceived Availability of Social Support, PHQ: Patient Health Questionnaire: SEASA: Self-Efficacy to Avoid Suicidal Action.

**Fig 2:**
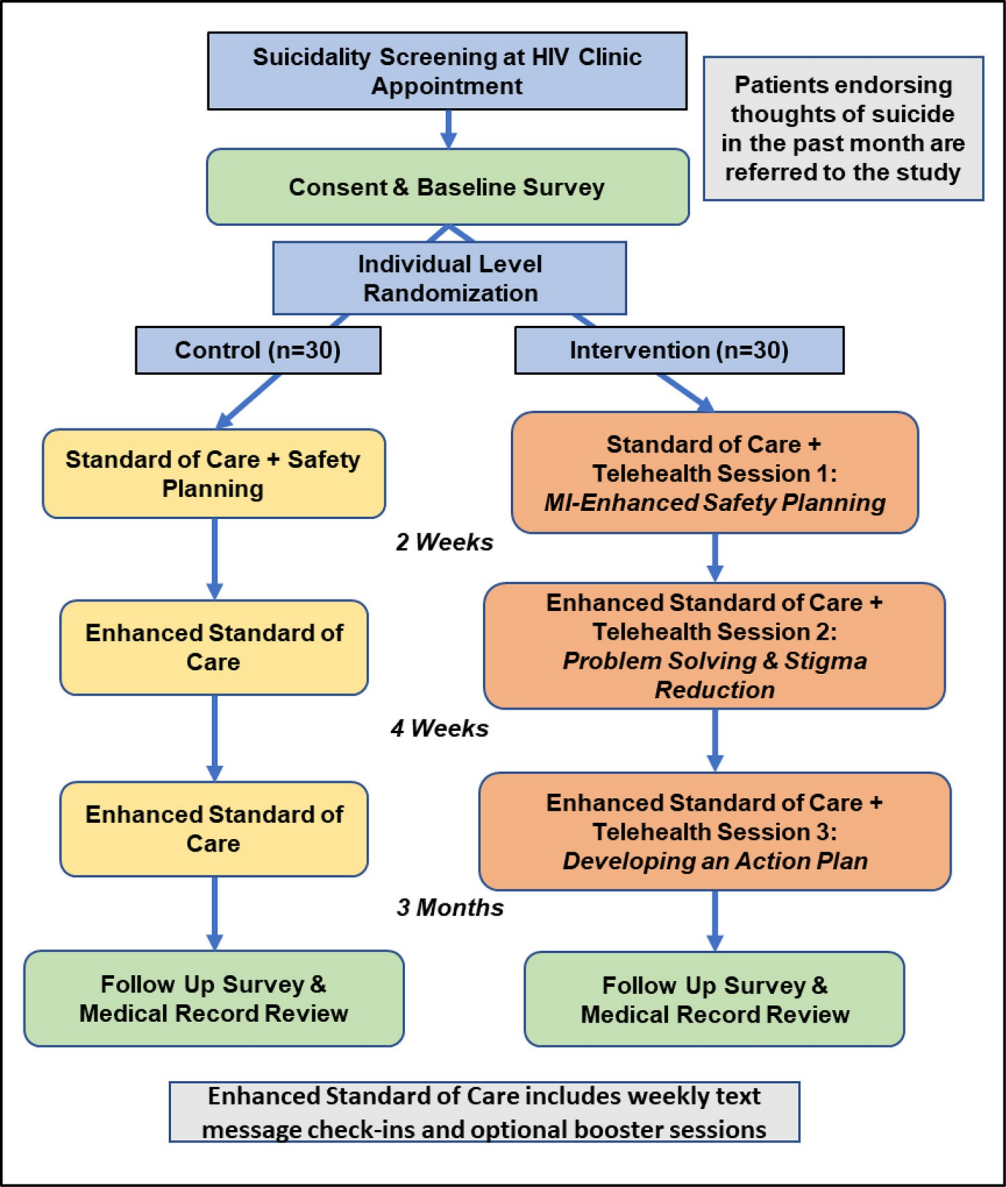
Flow diagram.

Patients who respond yes to the suicide screening question, are ≥ 18 years old, and speak either Kiswahili or English will be invited to enroll in the study. Those who respond yes but are otherwise ineligible for the study or who decline to participate will be referred for standard of care psychiatry services. After enrollment, participants will complete a baseline survey and will then be randomized 1:1 into the intervention or a comparison condition. Given the heightened risk of suicide in the study population, it was determined that participants in both the intervention and comparison groups should receive more than the existing standard of care in this setting. Participants assigned to the intervention cohort will receive the three-session telehealth-based IDEAS for Hope intervention with the first session on the day of enrollment, immediately after randomization. The comparison cohort will receive a single, brief telehealth-based safety planning session, also delivered on the day of enrollment, immediately after randomization. Participants in both conditions will be assessed for suicide risk at the conclusion of each contact, will receive a referral for psychiatric care, and will be directly accompanied to the psychiatry service if they have an active plan or intent to attempt suicide. Baseline data will be collected at enrollment and follow-up data will be collected at three months post-enrollment via researcher-administered survey and medical record review. A SPIRIT schedule of enrollment, interventions, and assessments is provided in Fig 1 and a flow chart of anticipated participant progress through the study is shown in Fig 2.

### Ethical approval

This study received ethical approval from the Duke University Medical Centre IRB (Pro00107424), the Kilimanjaro Christian Medical Centre IRB (Protocol 1307), and the Tanzanian National Institute for Medical Research (NIMR) (Protocol NIMR/HQ/R.8c/Vol.I/2122). All participants will provide written informed consent at the time of enrollment.

### Study setting

Participants will be recruited from two health facilities located in Moshi, Tanzania: Mawenzi Hospital and Majengo Health Center. Patient screening will be conducted by clinic nurses during regular HIV care appointments in the adult clinics at each health center, known as Care and Treatment Centers (CTCs). Patients identified as experiencing suicidal ideation during screening will be informed of the research study and, if interested in participating, will be escorted to a private office in the clinic to complete written informed consent, the researcher-administered baseline survey, and randomization. After the survey and randomization, they will be connected by a WhatsApp video call on study-owned tablets to meet with a telehealth counsellor (THC) located at the telehealth hub at a third site in Moshi, Kilimanjaro Christian Medical Center (KCMC) for either Session 1 of IDEAS for Hope or the comparison condition safety planning session. The two THCs will both be Diploma-level nurses trained to deliver the sessions according to the process detailed in the “Quality assurance” section below.

Collectively, the adult CTCs for HIV at Mawenzi and Majengo test over 300 patients for HIV each month and provide HIV care for over 6,000 PLWH within the region. Each facility follows national protocols for HIV testing and treatment, whereby all PLWH seeking care receive HIV care and medication free of charge. As part of the current standard of care, people experiencing emotional distress receive some brief counseling provided by nurses during HIV care. However, a prior study in this setting identified that HIV nurses have little formal training in mental health care and feel that they do not have adequate time to provide counseling to patients in distress [25].

With regard to specialized mental health care, KCMC has a psychiatry service for assessment, outpatient treatment, and medication management; Majengo has an on-site counselor to provide assessment and counseling; and Mawenzi is the site of the only inpatient psychiatric unit in the region, offering assessment, counseling, and both inpatient and outpatient treatment and medication management. However, our formative work identified that prior to this research, referral pathways from the HIV clinics to psychiatric services were not well established [31].

### Participants

A total of 60 PLWH who attend a standard HIV appointment at Mawenzi or Majengo and who respond affirmatively to the suicide screening question will be enrolled in the clinical trial. The sample size for this pilot trial was selected based upon the number of participants likely to be enrolled during the one-year study period using estimates from a prior validation study [31]. As such, the sample size is not intended to power statistical analyses of participant suicidality or care engagement outcomes.

### Screening and recruitment

In the waiting rooms at the Mawenzi and Majengo CTCs, a clinic nurse and study research assistant (RA) will provide a brief introduction of the study in Kiswahili to patients awaiting standard HIV care. Then, during their HIV care appointment, the nurse or doctor will administer the brief screening form, consisting of the single suicide screening question and the Patient Health Questionnaire-2 for depression (S1 Appendix). Patients who screen positively for depression but not suicidality will not be eligible for the clinical trial, but will instead complete a brief module of problem solving therapy based on WHO’s Problem Management+ [32] with the THC located at the telehealth hub. Patients who screen positively for suicidality but do not meet other eligibility criteria or decline to participate will receive a referral for psychiatric treatment, which is the current standard of care.

Patients who screen positively for suicidality and who are eligible and interested to learn more about the clinical trial will be referred to the study RA for confirmation of screening and inclusion criteria, and to provide written informed consent. Participant tracking information, contact preferences, and emergency contact details will be collected using a ‘Contact Information Sheet’ (S2 Appendix). The study RA will notify the THC of the newly enrolled participant and assign a unique participant identification (ID) number based upon site of enrollment so that all participant data can be de-identified. The RA will then verbally administer a structured baseline survey and enter participant responses directly into the Research Electronic Data Capture (REDCap) mobile application using a tablet. The RA will ensure the participant is comfortable throughout the survey and will pause if they are showing signs of fatigue or emotional distress.

### Survey measures

Baseline data collected will include demographic variables, HIV history, treatment adherence, HIV serostatus disclosure, measures of depression, anxiety, suicidality, HIV care engagement, HIV stigma, perceived social support, quality of life, and motivation for counseling. All study instruments were previously translated and used in prior studies in Tanzania [21, 31, 33] We reevaluated the accuracy of these prior translations for linguistic and cultural equivalence through a team-based process, whereby bilingual members of the research team translated and back-translated the items and discussed potential edits with the team until consensus was reached.

#### Sociodemographic variables

We will collect a variety of sociodemographic and participant history variables, such as the participant’s age, gender, relationship status, education, employment, religion, and distance from home to the clinic. Socioeconomic status will be measured by household income and questions related to the participant’s living situation, such as whether they have electricity, indoor plumbing, or a television in their home. We will also ask about food insecurity and housing insecurity using a modified version of the Household Food Insecurity Access Scale, which has been previously validated in Tanzania [34].

#### HIV and mental health history

HIV history will be collected with questions related to diagnoses, time from diagnosis, prior care engagement, and medication regimen. Antiretroviral medication adherence will be measured with 14- and 90-day self-reported recalls of missed medication doses. HIV disclosure will be measured with questions about whether the participant had disclosed their HIV status to a romantic partner, family, friends, or others. Mental health history will be measured with questions about whether the participant had sought counseling or support from various sources, taken medication for a mental health condition, or been diagnosed with a mental illness. HIV and mental health care engagement will also be recorded via a medical record review at the time of the 3-month follow-up.

#### Mental health symptoms

Participant suicidality will be assessed using the C-SSRS Screen Version for severity of risk [29], the Self-Efficacy to Avoid Suicidal Action (SEASA) Scale [35], a modified brief version of the Beck Hopelessness Scale [36], and the Brief Reasons for Living Inventory [37]. We recently validated these suicide measures with a sample of PLWH experiencing suicidal ideation in Tanzania and an associated manuscript is currently under review. Depression will be measured with the Patient Health Questionnaire-9, which was previously validated in Tanzania [38]. Anxiety will be measured with a modified anxiety subscale of the Brief Symptom Inventory [39].

#### Other potential covariates

We will also measure attitudes toward taking antiretroviral medication with the Beliefs About Medicines Questionnare [40], HIV acceptance with an adapted Illness Cognition Questionnaire [41], experiences of HIV stigma with an adapted short form of the HIV Stigma Scale [42], and social support with the Perceived Availability of Social Support scale [43]. Quality of life will be measured with two items from the World Health Organization’s brief Quality of Life Assessment [44].

#### Participant satisfaction with the intervention

On the 3-month follow-up survey, we will assess the participant’s satisfaction with the overall intervention, its format, the counselor, and various intervention components with questions on a 4-point Likert-type scale ranging from strongly disagree to strongly agree. We will also ask six open-ended questions to obtain qualitative feedback on helpful aspects, unhelpful aspects, influence on feelings about HIV, influence on mental health, influence on motivation to engage in HIV care, and any other feedback.

The full baseline and 3-month surveys can be found in S3 Appendix. The final question of the baseline survey will prompt the researcher to initiate allocation into the intervention or comparison condition.

### Allocation

Allocation sequences will be prepared by statisticians at the Duke Global Health Institute’s Research Design and Analysis Core prior to commencing recruitment. Participants will be randomized into the intervention or comparison condition in a 1:1 ratio using a stratified block randomization method, whereby SAS analytics software will be used to produce 4-person blocks within each of four strata defined by clinic and sex of the participant (2x2). Two stacks of sealed, numbered envelopes (one for women and one for men) containing the assigned allocation cohort will be prepared for each site. The RA will be blinded to the allocation sequence in advance. When prompted by the baseline survey, the RA will open the top envelope of the relevant stratum to reveal the cohort to which the participant has been allocated. The RA will inform the participant, enter the assigned allocation into the baseline survey, and inform the THC of participant allocation via text message.

### Intervention and control procedures

Immediately following randomization, the RA will provide a private space in the study facility and assist with the participant to make a WhatsApp video call on a study-owned tablet to connect with the THC. If requested by the participant, she/he may be accompanied by a treatment supporter such as a family member or partner during the session. Otherwise, the participant will be given privacy to carry out the call. The THC will first administer a ‘Pre-Counselling Survey’ of current symptoms (S4 Appendix). The THC will then commence either the brief (approximately 20 minute) safety planning module or the first of three telehealth intervention sessions. The THC will conclude the session by conducting a ‘Post-Counselling Survey’ to re-assess current symptoms and determine participant risk for suicidal action (S4 Appendix).

The RA will provide all participants (intervention and comparison condition) with a ‘Resources Sheet’ with contact details for the THC, psychiatry referral, and after-hour crisis resources, and will encourage the participant to enter these details into their phone. The RA will inform participants that they are welcome to contact the THC for optional counseling ‘booster sessions’ or reach out by text message during normal business hours for the duration of the study.

If the participant demonstrates signs of distress during the session, the session may be paused, postponed, or terminated. When active risk is detected (i.e., the participant endorses active plan and/or intent to attempt suicide), the THC will discuss with the participant the need for additional treatment and will inform the RA that a direct handoff to psychiatric care is needed. The RA will then contact the counselor on-site at Majengo or a counselor from the psychiatric unit at Mawenzi to coordinate a direct handoff for additional support. If a participant at risk is unwilling to attend psychiatry services, the HIV clinic nurses will be notified, and the patient’s emergency contact will be called to come to the clinic and assist.

After the first session (or sole session, for the comparison condition) participants will receive two check-in text messages from the THC at one and three weeks following the session. Based on feedback we received during our formative work, the text message will use vague language checking in on the participant’s well-being and will not include names of the researcher or clinic, nor reference to mental health or HIV.

For participants randomized to the intervention cohort, the two remaining telehealth sessions will be scheduled at two and four weeks following the first session. Participants may choose to complete these telehealth sessions via video call at the clinic, via video call at a location of their choosing, or via a regular phone call at a location of their choosing. The RA will ensure that scheduled dates of each session, check-ins, and the follow-up survey are noted on the participant’s ‘Resources Sheet.’ Reminders will be sent to participants prior to each session via their recorded outreach preference. If a session is missed, the THC will attempt to contact the participant according to their recorded outreach preference. Participants may withdraw their consent from the study at any time. If a participant no longer wishes to take part in the research, this will be documented, and contact will immediately cease. Prior data collected from participants who withdraw will be used unless the participant asks for it to be removed, in which case it will be deleted and the rationale will be documented. The ‘Pre- and Post-Counselling Surveys’ will be administered at each session and further care will be arranged as required.

Participants will receive 10,000 TZ shillings (equivalent to roughly $4.50 U.S.) for each in-person or video call session and 5,000 TZ shillings ($2.50) for each voice call session. Rates of compensation are in line with regional norms, and take into consideration transportation and mobile data/calling costs. The difference in compensation for in-person/video calls versus voice calls is due to the fact that travel to the clinic and mobile data incur a cost, whereas receiving a voice call does not. Participants who attend in-person will receive cash payment, while those who receive a call will receive a mobile electronic payment, which is a commonly used form of monetary transfer in this setting.

All problem management, intervention, and comparison condition sessions will be audio recorded using a digital recorder located in the telehealth office, transferred by USB to a secure online database, and then deleted from the recorder. Responses to the ‘Pre- and Post-Counselling Surveys’ will be entered directly into the REDCap application during administration. THCs will document key aspects of each session, including how risk was assessed, when risk occurred, and how it was addressed, within 24 hours using the ‘Counselling Documentation Form’ (S5 Appendix) in a data, action, plan format. Counseling notes will be saved on a secure electronic database managed by Duke University. Further details on the problem management session, the control safety planning module, and the three telehealth intervention sessions are provided below.

### Non-enrolled patients with symptoms of depression only: Problem management session

The THC will administer the pre-counselling survey prior to commencing the problem management session. The THC will begin the session by introducing its purpose and guiding the participant to provide three current problems they are facing and to identify a solvable aspect of a problem they wish to address. Collaboratively, the THC and participant will break down the problem and define when it began, situations in which it occurs, how the participant responds emotionally, the consequences, and the potential impact of removing the problem. The THC will help and encourage the participant to identify two or three solutions and to build a step-by-step plan for implementing these solutions. Once the session is concluded, the THC will administer the post-counselling survey and will contact the RA to return to the participant. If acute distress is identified during the session or post-counselling survey, the RA will be notified to assist with a direct referral to psychiatric care. If no acute distress is identified, psychiatry contact information will be provided. The cost of a first psychiatry session will be covered by the study.

### Comparison condition: Brief safety planning session

The brief safety planning session, adapted from the Stanley & Brown safety planning intervention, has been translated to Kiswahili and adapted by the study team to ensure cultural, linguistic, and contextual relevance and sensitivity for use in Tanzania [45]. This session aims to familiarize participants with their personal warning signs of a suicidal crisis to allow them to initiate coping strategies before escalation. The THC will begin by encouraging the participant to identify three personal coping strategies to use when feeling suicidal, as well as three people in their personal lives who they feel can provide support. The THC will also assist the participant with identifying professionals whom they may contact during a crisis. Hospital counsellors will be suggested to the participants for this purpose and their contact details will be provided. Steps to improve the safety of the participant’s home environment (such as removing lethal means) will be discussed. Finally, the THC will encourage the participant to discuss their personal reasons for living and wanting to stay safe.

### IDEAS for hope intervention condition: Three sessions of telehealth-delivered counseling

The conceptual model for intervention development is the Consolidated Framework for Implementation Research (CFIR), which describes intervention characteristics; the outer setting, assessing the compatibility of the intervention with the realities of the health system; and the inner setting, including reactions of participants during the clinical trial [46]. The CFIR conceptualizes interventions as being made up of stable core characteristics, in this case the theoretical frameworks informing intervention development, and an adaptable periphery, including the length, number, format, spacing, and location of sessions. A breakdown of intervention characteristics and implementation strategies are provided in Table 1.

**Table 1:** Characteristics and implementation of the telehealth intervention, informed by the CFIR.

Intervention Characteristics	Inner Setting	Outer Setting	Implementation Process
• Past intervention models: MI-SafeCope, Problem Solving, and CBT-AD to address the unique burden of HIV • Brief format for feasibility in low-resource setting • Telehealth delivery to enhance reach	• Need for mental health support in HIV care • Universal screening to improve symptom recognition • Integration with existing clinical services • Referral of PLWH with suicidal ideation for higher level of care	• High burden of depression and suicidality among PLWH • Growing public awareness, support for mental health treatment • Benefit of fostering family and social support for treatment	• Involvement of local partners in intervention design in formative work • Close collaboration with clinic nurses for uptake of screening and referral • Communication with hospital and government leaders
**Individuals Involved**			
• Telehealth counselor • PLWH • Nurses and on-site psychiatric services	• Clinic nurses • Mental health workers • Hospital administrators • Government officials	• PLWH experiencing suicidal thoughts • Family, friends, partners • Community groups	• Mental health workers • Clinic nurses • Hospital administrators • Government officials
**Intervention core characteristics:** MI-enhanced screening and safety planning, CBT-AD to improve cognitive flexibility for care engagement, education to reduce misinformation and stigma; problem solving to improve social support.			
**Adaptable periphery:** Delivery format (telehealth), intervention delivery by non-mental health specialist counselor, length, number, format, spacing, and location of sessions, cultural and linguistic adaptation for Tanzanian context.			

PLWH: people living with HIV (Knettel, 2020, used with permission).

**Fig 3:**
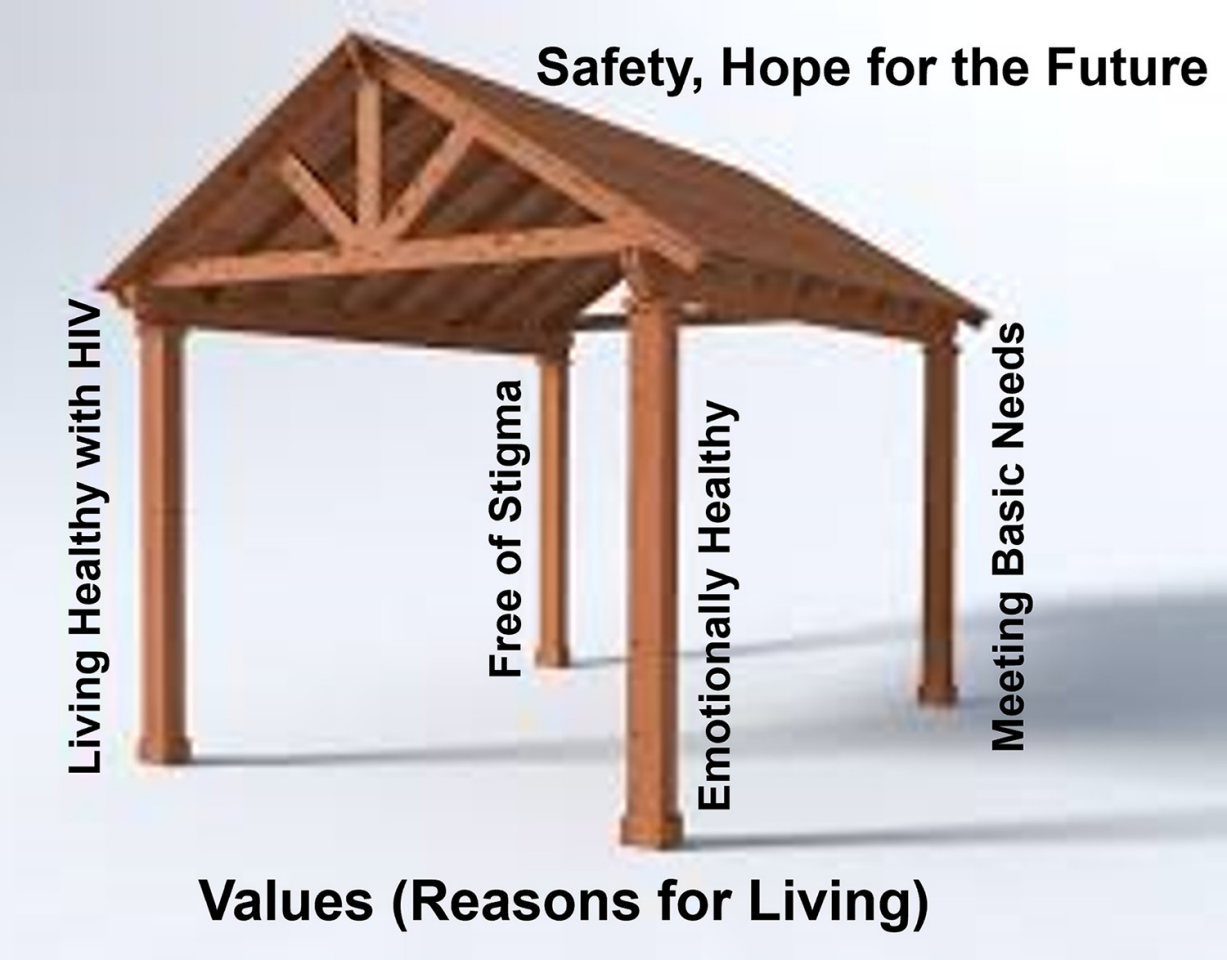
The four pillars framework. (Knettel, 2023, used with permission).

The three telehealth intervention sessions will occur at two-week intervals and will incorporate intervention strategies represented by the four pillars, foundation, and roof in the Four Pillars framework: 1) provide education and dispelling myths to support living healthy with HIV, 2) develop coping skills to combat HIV stigma through storytelling, 3) behavioral activation to seek social support and utilize counseling services, and 4) address basic needs with problem-solving skills. These four pillars support the roof, which represents safety and hope for the future, practiced through MI-enhanced safety planning. All of the activities are informed by the foundation of unique personal values and reasons for living identified early in the intervention (Fig 3). The first and second sessions are expected to take approximately 60 minutes and the third session is expected to take approximately 20 minutes.

#### Session 1 –MI-enhanced safety planning

During the first session, the telehealth counselor will introduce the Four Pillars framework and use MI to learn about the participant’s history and challenges with HIV and suicidal thinking. Conducted with a focus on compassionate active listening and counseling skills, this “HIV and mental health journey” assists in building rapport while also identifying the participant’s personal values and reasons for living. The THC will use MI to identify and develop discrepancies between suicidal actions and personal values, such as health, spirituality, and family relationships.

Regarding barriers to HIV care engagement, the THC will guide the participant to discuss and dispel common myths and misinformation relate to HIV, including the belief that HIV inevitably leads to early death, that treatment is burdensome and expensive, and that they will inevitably transmit to their sexual partners. When misinformation is present, HIV education will be provided, such as information that an undetectable viral make it impossible to transmit HIV to a partner (known as undetectable = untransmittable or U = U) [47]. The THC will also explore values supporting HIV disclosure, concerns regarding transmission, and strategies for coping with HIV stigma. Using MI, the THC and participant will identify parallels between concepts of hope for the future and the physical and emotional well-being associated with an undetectable HIV viral load, which can lessen the burden of HIV. The THC will emphasize the possibility of living a long life according to personal values, free of symptoms and safe from transmitting the virus.

Next, the safety plan component of MI-SafeCope, adapted with HIV stigma and adherence frameworks [6, 48], will be delivered to target unique contributors to suicidal ideation and risk among PLWH, including lack of knowledge, misinformation, and fears related to deteriorating health, HIV disclosure, and concerns about transmission [26]. Collaboratively, the THC and participant will identify personal coping strategies for suicidal thoughts, informed by personal values, and external sources of support including friends, family, and health professionals. The THC will also assist the participant with creating a plan and goals for eliciting support. Finally, the THC and participant will devise strategies to improve safety at home, such as removing access to lethal means. The session will conclude by describing a hopeful future in revisiting the concepts of personal values and reasons for living.

#### Session 2 –Problem solving and stigma reduction through storytelling

The second session of IDEAS for Hope will be guided by syndemic social determinants theory [49] and the HIV Stigma Framework [6] to target stigma (internalized, enacted, and anticipated) as a key contributor to suicidality. The THC will initiate a discussion surrounding stigma with a storytelling vignette about an individual who was diagnosed with HIV, experienced the various forms of stigma, struggled with suicidal thoughts, and was ambivalent about care engagement, but then utilized resources and achieved a sense of recovery [28]. The concept of recovery is critical to the stigma reduction component, as it emphasizes that challenges associated with HIV and mental health can be overcome.

Aspects of cognitive behavioral therapy for adherence and depression (CBT-AD) are also integrated, including acknowledgement of hopelessness driven by expectations of the worst outcome, which can be restructured through normalization and personalization in a guided, supportive discussion of the storytelling vignette [48, 50]. The THC will describe positive examples of social support for PLWH and the known waxing and waning of associated symptoms of distress to emphasize the potential for long-term recovery from symptoms. Participants will be encouraged to participate meaningfully with HIV care engagement content by drawing parallels with their own experiences, challenges, and goals for the future.

In the second half of the session, the counselor introduces the problem-solving framework for meeting basic needs. In our formative work, social challenges such as poverty, food insecurity, and housing insecurity were key drivers of suicidal thinking. Problem-solving therapy involves breaking larger problems into smaller, more manageable parts and identifying aspects of the problem that are within one’s control and can benefit from problem-solving [51]. Through problem-solving, the participant is empowered to take ownership in her/his own recovery, and to begin making changes toward improvement, which are integrated into a structured plan in Session 3.

#### Session 3 –Developing an action plan for maintaining safety and HIV care engagement

During the third session, attention will shift to leveraging problem solving to develop a structured action plan for ongoing improvement. The THC will revisit the Four Pillars framework, reviewing content and progress in the areas of improved HIV knowledge, strategies for coping with stigma, enhanced social support, and meeting basic needs. Through this process, the THC and the participant will identify problems that feel unresolved, break down those problems into aspects that are solvable, brainstorm solutions, and develop a structured action plan for addressing the problems. The third session will conclude with a summary of key points from the three sessions, review of the action plan, and expressions of thanks and hope for the future.

### Participant follow-up

Participants will be asked to return to the clinic at three months post-enrollment to complete the 3-month follow-up survey (S3 Appendix). The survey will be verbally administered by the RA. When in-person follow-up is not possible, phone calls will be arranged. The follow-up survey will repeat the measures collected at baseline to assess for change during the study period, as well as new measures of intervention satisfaction, barriers and facilitators to study participation, and feedback to inform intervention refinement (Fig 2). The RA will also perform a review of medical records at the three-month time point to collect data on mental health care engagement and to corroborate self-reported HIV clinic attendance. Participant names will be used to obtain information from paper charts and electronic medical records, and will be securely stored according to approved IRB protocols clearly described in the informed consent form.

### Monitoring

Adverse events may occur during the study due to the high-risk nature of working with people experiencing suicidal ideation. However, these events are not anticipated to be related to study participation or randomization due to the low-risk nature of counselling interventions. The study team will continually monitor safety indicators, including suicide attempts and suicide fatality, and all adverse events will be reported to the sponsoring institutions, funder, and review boards according to their respective policies. In the unlikely event of increased suicidality within the intervention cohort compared with the comparison condition, the trial will be discontinued.

### Quality assurance

All clinic nurses and the nurse in-charge at Majengo and Mawenzi and all study RAs will receive training in the following: introduction to the ‘Screening Form’ and procedures for screening, monitoring the clinic log for screening fidelity, approaches to avoid stigmatizing HIV and mental health (e.g., use of respectful, non-stigmatizing language), engaging with vulnerable populations, basic counselling skills, ethical principles, and strategies to minimize emotional distress. All study staff will receive training in suicide assessment and safety planning.

THCs will receive training in counseling skills, the intervention model, and ethical/safety training via three weeks of didactic and mock intervention sessions. The quality of counseling sessions will be assessed via the Therapy Quality Scale (TQS), which includes ratings of fidelity (completion of core intervention components), quality of intervention pieces, and counseling skills (e.g., eliciting hope, active listening). Fidelity items will be rated as 0 –“Not Done”, 1 –“Partial”, or 2 –“Completed” for a total score of 0 to 10. Quality and counseling skills items will rated on a scale of 0 –“Not Done” to 4 –“Excellent”. Acceptable fidelity is defined as the mean of the fidelity items exceeding a threshold of 1.8 or greater (out of 2 possible, indicating 90% completion of core assessment components). For quality and counseling skills items, we will use a pre-established threshold of item means of 3 or greater (out of 4 possible, representing “Good” to “Excellent” quality or skills) as acceptable. Interrater reliability will be measured, and discrepancies > 1 point will be discussed to improve the rating process.

Throughout the study, these data will be continually reviewed to support counselor skill development and identify emerging training needs. Upon commencement of the clinical trial, THCs will receive weekly clinical supervision with a psychiatrist and a psychologist, including reviews and feedback of audio recorded counselling sessions. Sessions will be rated using the TQS to assess ongoing counseling skills and fidelity and additional retraining will be provided as appropriate.

### End of day tasks and data management

At the end of each day, the RA will collect all ‘Screening Forms’, cross-check screening with clinic attendance logs, and conduct a review and update of the ‘Tracking Log.’ The number of patients seen daily will be recorded in the ‘Tracking Log’ with explanations for discrepancies identified between the number of patients screened and number of patients seen. Patients who screen positively for suicidality, but are deemed ineligible or decline to participate, will be documented in the ‘Screening’ tab under the ‘Number Screened Positive for SI but Not Enrolled.’

Signed informed consent forms and patient contact information forms will be stored in a locked cabinet at each enrolling clinic during the week and transferred by the RA to a secure storage room at Kilimanjaro Clinical Research Institute at the end of each week. The key for participant ID numbers will be stored within secured files on password-protected computers. The RA will transfer data from voice recorders to storage on secure electronic servers each day and will then delete the recording from the voice recorder. The data analyst will exclusively analyze de-identified data and will ensure that access to electronic and physical data is restricted to key study personnel only.

### Outcomes and data analysis

*Feasibility and acceptability* of the intervention will be described by retention and session attendance patterns, qualitative data exploring the experiences of participants, and data from the 3-month survey. *Participant satisfaction* will be measured using the Client Satisfaction Questionnaire [52] at the 3-month survey and will be defined as acceptable if a mean score of ≥3 is reached out of a possible 4. *Fidelity and quality* of the intervention will be measured by the TQS and deemed acceptable if mean scores are ≥ 3 on a 0–4 scale.

*Qualitative feedback* will be analyzed using Applied Thematic Analysis informed by Grounded Theory [53, 54], a rigorous approach to hypothesis testing with textual data in a way that is transparent and reproducible. Interview transcripts will first be summarized in a qualitative memo, including representative quotes, and organized around a priori domains reflecting the components of the interview guides and the CFIR. Next, the memos will be uploaded to NVivo software for coding. Within the broader domains, emerging themes from the interviews will be identified inductively and added to a codebook to develop a coding structure. Each memo will be coded by two team members, inter-coder agreement will be calculated, and disagreements will be reconciled via consensus discussion with a third team member. After coding, we will produce NVivo queries and analytic memos to synthesize content, compare participant characteristics, and draw deeper meaning on themes.

The potential efficacy of the intervention will be assessed by analyzing primary and secondary outcome differences between the control and intervention groups. The primary outcomes of the study are severity of suicidal ideation and HIV care engagement (self-reported adherence to ART, clinic attendance). Preliminary statistical analyses will assess factors associated with severity of suicidal ideation and HIV care engagement at baseline. The main analysis will assess the influence of assigned intervention condition and other baseline factors associated with the primary outcomes at three months of follow-up. Secondary outcomes in both preliminary and main analyses will include depression, HIV stigma, HIV acceptance, social support, and quality of life.

An intent-to-treat model will be used, whereby all participants who are randomized are included in the statistical analysis and analyzed according to the group they were originally assigned, regardless of what treatment they receive. Loss to follow-up at the three-month time point will be compared between the study arms. The trial will not be powered to detect significant intervention effects; however, these analyses will be used to generate parameter estimates and ranges of values for design of a larger future trial. Mixed-effects regression will be used to model outcome differences within and between study arms using a time by condition model. Individual-level random intercepts will account for counselor affects and correlation due to repeated measurement. Analysis will be carried out using SAS software.

### Study organization

BAK (Duke University) and BTM (KCMC) are the principal investigators of this study and will be responsible for the integrity and completion of this work, as well as facilitating communication between Duke University and KCMC. The principal investigators will be responsible for data analyses, with assistance from CAS, DBG, and MVR of the mentorship team, along with support from ELT and JRNV of the Duke Global Health Institute Research Design and Analysis Core. EFM is the study coordinator and will oversee all study activities. IA was the prior study coordinator and is currently a member of the Duke support team. ETK is a clinical research coordinator at Duke University. VK and CST are RAs based at the Majengo and Mawenzi sites, respectively. JB and KM are clinical consultants and psychiatrists at the KCMC Department of Psychiatry who will assist with clinical supervision of the THCs and will facilitate referrals to standard of care at KCMC. LJ and JR are nurse-counselors at the KCMC telehealth hub.

### Limitations and strengths

This study has several limitations. Participants enrolled will reflect those who actively seek HIV care and thus may not represent the full population of PLWH within the community in this region. This study also relies on participant-reported suicidal symptoms, which participants may not wish to disclose. Finally, the study is not powered to test efficacy; therefore, analyses to this effect must be considered preliminary. However, this study has strong potential to inform future interventions that will provide much-needed support for the mental health of PLWH in a severely under-resourced setting. The intervention and clinical trial have been thoughtfully designed, in collaboration with local partners and using the CFIR implementation framework, to increase the likelihood of successful integration into the health system.

## Discussion

The IDEAS for HOPE trial will provide the first evidence-based data for a telehealth intervention for suicidality and HIV care engagement in Tanzania. This intervention uniquely addresses the specialized needs of PLWH in low-resource settings by merging components from two theoretical frameworks—MI-SafeCope and Problem-Solving Therapy—to enhance HIV care engagement, reduce stigma, meet basic needs, and foster hope among PLWH [9, 14, 15, 26–28, 51]. Once safety has been established, the intervention is designed to treat underlying drivers of hopelessness and enhance HIV treatment adherence through HIV education, stigma reduction, problem-solving, and enhancing social support. The MI-SafeCope model is a promising intervention which has demonstrated positive outcomes for developing suicidality coping strategies, through individualized safety plans, facilitating social support, and a telephone-based session [26]. A combination of strong HIV-focused counseling with the crisis-based MI-SafeCope model of suicide prevention has the potential to provide a condensed and focused strategy to reduce suicidality and enhance feelings of hope in low-resource settings.

This pilot feasibility trial will assess opportunities for intervention integration into HIV care in Tanzania. The intervention has strong potential for future scale-up within the Kilimanjaro region and may be adapted to other settings in the future, as it utilizes pre-existing healthcare infrastructure and minimizes the burden placed on already overtaxed healthcare systems. Thus, results from this trial, although not powered to statistically analyze effectiveness, will be used to inform a future randomized clinical trial of a larger population of PLWH, should the intervention prove feasible.

## Supporting information

S1 Checklist SPIRIT checklist. (PDF)

S1 File Approved study protocol. (PDF)

S1 Appendix Screening form. (PDF)

S2 Appendix Contact information form. (PDF)

S3 Appendix Study surveys. (PDF)

S4 Appendix Pre- and Post-counselling survey. (PDF)

S5 Appendix Counselling documentation form. (PDF)
