# Supplementary material for: Protocol for a pilot randomized controlled trial of a telehealth-delivered counseling intervention to reduce suicidality and improve HIV care engagement in Tanzania
Source: PLoS One. 2023 Jul 27;18(7):e0289119. doi: 10.1371/journal.pone.0289119 (PMC10374000; doi:10.1371/journal.pone.0289119)
Supplement: S1 File — (PDF) [file pone.0289119.s002.pdf]

TITLE: **Informing, Designing, and Testing a Telehealth Intervention to Address HIV and Cancer-Related Stigma in Tanzania**

Protocol: Version 3
Date: 30 August 2021

Principal Investigators:

Local Principal Investigator: Prof. Blandina T. Mmbaga

Foreign Principal Investigator: Dr. Nosayaba Osazuwa-Peters

Foreign Principal Investigator: Dr. Brandon Knettel

Other investigators:

Local Investigators:

Dr. Judith Boshe

Ms. Elizabeth Msoka

Ms. Linda Minja

Funding Agency: Duke Global Health Institute, U.S. National Institute of Mental Health (NIMH)

Project Duration: Four Years

Table of Contents

List of acronyms ii

Summary: 3

Introduction and Literature Review: 3

Statement of the Problem: 3

Rationale: 4

Objectives: 4

Methodology: 5

Objective 1: In-depth Interviews with stakeholders 5

Objective 2: Cross-sectional survey 5

Objective 3: Intervention development 6

Objective 4: Pilot clinical trial of counseling intervention 6

Risks and Benefits: 8

Confidentiality: 9

Informed consent: 9

Budget: 13

References 11

Appendices 14

List of acronyms

ART	Antiretroviral therapy
CBT-AD	Cognitive Behavioral Therapy with Adherence Counseling
CTC	Care and treatment center
CFIR	Consolidated Framework for Implementation Research
DGHI	Duke Global Health Institute
HIV	Human Immuno-defieciency Virus
IDI	In-depth Interview
KCRI	Kilimanjaro Clinical Research Institute
KCMC	Kilimanjaro Christian Medical Centre
MI	Motivational Interviewing
PhD	Doctor of Philosophy
PLWH	People living with HIV
RCT	Randomized controlled trial
SoC	Standard of care

Summary:

Stigma is a key driver of the global disparities in HIV and cancer care, contributing to substandard care engagement, reduced quality of life, and lower life expectancy for both diseases in low- and middle-income countries, including in Tanzania.

The proposed research will address the critical need to reduce stigma and support engagement with care after receiving a life-changing diagnosis of HIV or cancer, and therefore support the success of treatment at Tanzanian hospitals and clinics.

First, we conduct formative qualitative research to understand stigma as a contributor to substandard care engagement and associated mental health challenges, including suicidal ideation. Methods will include in-depth qualitative interviews with patients (n=20), health center administrators (n=5), clinic nurses (n=7), and mental health workers (n=7).

Second, we will translate from English to Swahili language several common measures of stigma and emotional distress common to people living with HIV and cancer. We will then administer the measures to 80 patients to assess their validity.

Third, we will enroll a cross-sectional cohort of 150 clinically diagnosed cancer patients recruited from the outpatient clinic at Kilimanjaro Christian Medical Centre (KCMC) to better understand factors that influence stigma and the decision to seek cancer screening and treatment.

Fourth, we will develop and refine the telehealth intervention model with support from a study advisory board in Moshi. An advisory board of PLWH, nurses, and community leaders will provide input and feedback on the format, technologies, session content, level of structure, and length and timing of the sessions. The advisory board will have approximately 15 members and will convene 3 times during the study.

Fifth, we will conduct a randomized controlled trial (RCT) of a telehealth delivered counseling intervention for 60 people living with HIV (PLWH) and experiencing emotional distress, to examine feasibility and acceptability of the intervention, as well as its potential impact on stigma, mental health, and care engagement.

Background

The mortality burden of HIV and cancer in sub-Saharan Africa are remarkably high, with a disproportionate burden of HIV and cancer deaths coming from this region. In Tanzania, there are 1.6 million adults living with HIV, which is 4.6% of the population and the 5th highest incidence in the world.[1] There are over 750,000 new cases of cancer in Africa each year, and 500,000 cancer deaths.[2] Sadly, most cancer deaths are potentially preventable with effective screening, as the dominant cases are breast, cervical, and prostate cancers.[2]

In Tanzania, there are 35,000 new cancer cases annually, with 21,000 deaths, making it the nation's third leading cause of death.[3,4] However, there are only four cancer hospitals in the entire country, Aga Khan Hospital Dar-es-salaam, Bugando Medical Centre, Kilimanjaro Christian Medical Centre, and the Ocean Road Cancer Institute. Given the lack of care options, cancer is often considered a death sentence in Tanzania, and there is widespread fear and stigma associated with a cancer diagnosis.[5] Mitigating cancer stigma would drastically improve attitude towards screening, enhancing early detection and survivorship.[6]

Literature Review

Across multiple studies, emotional distress including depression and suicidal ideation has been linked to delayed initiation of treatment, poor care engagement, lower quality of life, and reduced life expectancy among people with both cancer and HIV.[7,8] However, when patients have access to evidence based mental health treatment that incorporates sound adherence counseling, cancer and HIV outcomes improve.[9–11]

Considerable efforts have been made to understand HIV stigma, such as through the HIV Stigma Framework of Earnshaw and colleagues[12], describing enacted, anticipated, and internalized forms of HIV stigma. However, to date, few interventions have successfully intervened to reduce HIV stigma and improve HIV care engagement[13], and fewer still have done so in a manner that can be effectively implemented and brought to scale in sub-Saharan Africa.[14]

The research on cancer stigma in Tanzania and other African countries is much more sparse, and there ample opportunity for new knowledge in this area.[15,16] For example, there is a clear need for studies that examine the translatability of HIV stigma frameworks to understand cancer stigma, care engagement, quality of life, and health outcomes.[17] Additionally, we must extend the stigma literature to capture the unique aspects of cancer stigma that may have been missed in previous, HIV-focused frameworks.[18]

The purpose of the current study is to extend existing frameworks for HIV stigma in Tanzania to better understand cancer-related stigma, and to develop and test a strong, scalable telehealth counseling intervention that may serve to address both HIV and cancer stigma in this country. The use of telehealth in the intervention will assist in overcoming human resource limitations in counseling.[19]

Kilimanjaro Christian Medical Centre (KCMC) and Duke Global Health Institute (DGHI) are committed to global health research and education focused on achieving health equity. Addressing HIV and cancer related stigma would fit this vision directly. The principal

investigators have a strong research background focused on addressing barriers to care engagement in Tanzania, including prior studies focused on HIV stigma.

Statement of the Problem

Given the strong impact of stigma, people often delay in seeking care for both HIV and cancer in Tanzania, contributing to thousands of unnecessary deaths each year. Mitigating HIV and cancer stigma through a targeted telehealth counseling intervention would drastically improve attitudes towards treatment, enhancing early detection, care engagement, and survivorship.

Main Objectives / Study Goals:

The main objectives of this study are to improve the understanding of HIV and cancer-related stigma in Tanzania, and to use this information to develop and test a telehealth counseling intervention in a randomized clinical trial.

Rationale:

This study will fill a critical void in understanding emotional distress and stigma in the context of HIV and cancer care, and will develop and test a brief telehealth counseling intervention to address these challenges. Using the Consolidated Framework for Implementation Research, this study will examine the current landscape of HIV and cancer care in the Kilimanjaro region, seeking varied perspectives (providers, patients) and using multiple methods (surveys, qualitative interviews, scale validation, pilot clinical trial) to inform and test the telehealth intervention model.

Objectives:

This study will address five objectives:

Objective 1: Identify the desired characteristics of a telehealth intervention for HIV stigma and care engagement. We will conduct in-depth qualitative interviews with people living with HIV (n=20), health center administrators (n=5), HIV clinic nurses (n=7), and mental health workers (n=7) to discuss potential benefits and barriers to a brief, telehealth-delivered counseling intervention in regional HIV clinics. Interviews will also assist in planning the structure of the telehealth counseling intervention planned for later in the study, including potential formats, technology, session content, level of structure, length and timing of sessions.

Objective 2: Translate and validate common stigma measures to ensure their applicability for both HIV and cancer stigma studies in Tanzania. We will undertake a rigorous procedure to translate common stigma scales from English to Kiswahili, with additional focus on ensuring that measures are culturally relevant to the Tanzanian context. We will administer the translated measures to 80 HIV and cancer patients in the Kilimanjaro region to evaluate their validity.

Objective 3: Conduct a survey of cancer patients to assess the applicability of existing stigma frameworks in the cancer population, and to understand the potential impact of stigma on treatment decision-making and outcomes. We will examine

predictors of treatment engagement, including self-reported cancer stigma, in a cross-sectional cohort of 150 cancer patients at KCMC using an adapted Cataldo Lung Cancer Stigma Scale (CLCSS). We will also determine associations between self-perceived stigma and timing of care-seeking. The main outcome of interest is time from onset of cancer-related symptoms to medical help–seeking for cancer symptoms.

Objective 4: Refine content for the telehealth intervention with support from a study advisory board in Tanzania. An advisory board of PLWH, nurses, and community leaders will assist in the development and refinement of the telehealth intervention. Advisory board members will be identified and invited to participate during in-depth interviews conducted for Objective 1. The advisory board will have approximately 16 members and will convene 3 times: 1) at the conclusion of Aim 1 to assist in refining the intervention prior to piloting by interacting with a prototype model, 2) midway through the clinical trial to review progress, and 3) at the conclusion of the trial to review and assist in interpreting findings. We will invite advisory board members to experience the intervention as a study participant would, by interacting with the intervention technology, counselors, and study surveys. The board will give feedback on the process and perceived effectiveness of the session. Through this process, we will reshape the intervention in real time and increase acceptability for both clinic staff and patients.

Using advisory board feedback, the study team will develop a detailed study protocol and intervention manual. The intervention will leverage existing Cognitive Behavioral Therapy-Adherence (CBT-AD) and Motivational Interviewing (MI-SafeCope) frameworks to address suicide risk and improve HIV care engagement.[20,21] Patients will be enrolled by study staff and will connect to the telehealth hub via mobile “hot spots” at study site clinics. Counseling will be provided by study nurses trained in the intervention model.

Objective 5: Pilot test the telehealth intervention in a randomized controlled trial to assess feasibility, acceptability, and potential efficacy for reducing suicidality and enhancing HIV care engagement. We will screen patients in HIV care in the Kilimanjaro region and enroll 60 PLWH who are experiencing suicidality to participate in a pilot feasibility trial. Participants will be randomized to receive (a) enhanced standard of care plus safety planning, or (b) the three-session telehealth intervention. Participants will complete assessments at baseline and 3 months post-enrollment exploring safety, acceptability, feasibility, and preliminary efficacy of the intervention.

Methodology:

Study Setting. The study will be located in health centers in Moshi, Tanzania: 1) Kilimanjaro Christian Medical Center (KCMC), a large tertiary hospital, 2) Mawenzi Hospital, a public facility housing the region’s only inpatient psychiatry unit, 3) Majengo Health Center, a small urban health clinic, 4) Pasua Health Center, a small urban health clinic, and 5) St. Joseph Hospital, a semi-public/semi-private hospital. KCMC and Mawenzi are the primary referral hospitals for the Northern Zone of Tanzania, covering an area of 122,000 square kilometers.[22] The adult Care and Treatment Clinics (CTCs) for HIV at these hospitals/clinics currently test more than 300 people for HIV each month and provide HIV care for more than 6,000 PLWH in the region. KCMC is home to one of only four cancer hospitals in Tanzania and the only cancer center in the Kilimanjaro region. Therefore, all cancer-related recruitment will occur at the Cancer Care Center at KCMC Hospital.

Objective 1: Identify the desired characteristics of a telehealth intervention for emotional distress, stigma, and HIV care engagement in the Tanzanian clinical context (Timeline: Late 2021, early 2022). We will conduct in-depth interviews (IDIs) with stakeholders including PLWH (n=20), clinic administrators (n=5), nurses (n=7), and mental health workers (n=7). For the patient interviews, clinic nurses will assist in identifying adults in HIV care at the study clinics who previously experienced suicidal ideation in the past year. All study staff will be trained in suicide assessment and safety planning. Administrators and health workers will be recruited from the study clinics.

Interview guides will be developed in collaboration with the Tanzanian research team and informed by CFIR domains. Participants will first be presented with the core characteristics of the intervention and asked to provide feedback. Interviews will also explore the outer setting of patient needs and resources and the relative advantage of the proposed intervention compared to other approaches. All participants will receive compensation of 10,000 Tanzanian shillings ($4.50) for their time and costs of participation. Interviews will be conducted in Kiswahili or English by a trained Tanzanian research assistant in a private office and audio recorded for later transcription.

Inclusion criteria for all participants will be: age 18 or older at the time of the interview and capable of understanding and providing informed consent to participate. Patients will be eligible if they have tested positive for HIV and indicate actual thoughts of suicide in the past month. Clinic administrators, clinical nurses, and mental health workers will be eligible if they oversee or provide clinical services related to HIV care and/or mental health in the Kilimanjaro region. Participants will be excluded if they are under age 18 or physically or cognitively not capable of completing the study procedures.

Qualitative data will be analyzed using a thematic approach based on grounded theory and the constant comparative method.[23,24] Emerging themes from the interviews will be developed into a codebook to identify a coding structure. Each memo will be coded by 2 team members, inter-coder agreement will be calculated[25], and disagreements will be reconciled via consensus discussion.

Objective 2: Translate and validate common stigma measures to ensure their applicability for both HIV and cancer stigma studies in Tanzania (Timeline: Late 2021, Early 2022). We will undertake a rigorous procedure to translate common stigma scales from English to Kiswahili, with additional focus on ensuring that measures are culturally relevant and valid in the Tanzanian context. Scales will include the Cataldo Lung Cancer Stigma Scale (CLCSS), HIV Stigma Scale (HSS), Beliefs About Medicines Questionnaire (BMQ), Illness Cognition Questionnaire (ICQ), Columbia-Suicide Severity Rating Scale (C-SSRS), Self-Efficacy to Avoid Suicidal Action Scale (SEASA), Beck Hopelessness Scale-Short Form (BHS-SF), and Brief Reasons for Living Scale (BRFL).

We will first ask a skilled bilingual translator to translate the measures from English to Kiswahili. Next, a second translator will back-translate the Kiswahili versions back to English, and the two versions will be compared for linguistic and cultural equivalence. The research team will then meet to reconcile the two versions and finalize the translated measures.

Once the measures are translated, we will administer them to 80 HIV and cancer patients at two hospitals/clinics in the Kilimanjaro region (KCMC and Pasua Health Centre) to evaluate

their validity. Of these, 20 participants will be asked to repeat the measures two weeks later to assess test-retest reliability. Clinic nurses will introduce the study to potential participants and those who are interested will be accompanied to a private study office to complete informed consent procedures. Those who agree to participate will complete the measures. Patients will receive 5000 Tanzanian shillings ($2.25) for participating.

Participants must be age 18 or older and capable of understanding and providing informed consent to participate. Patients will be eligible if they have been diagnosed with HIV or cancer and indicate actual thoughts of suicide in the past month. Participants will be excluded if they are under age 18 or physically or cognitively not capable of completing the study procedures.

The final data will be measured for internal consistency (Cronbach's alpha and item-scale correlation), convergent validity among related measures, and confirmatory factor analysis.

Objective 3: Enroll a cross-sectional cohort of clinically diagnosed cancer patients (Timeline: Late 2021, Early 2022). Participants will be recruited from the KCMC Cancer Centre in Moshi, Tanzania and asked to complete a one-time research survey in late 2021 and early 2022. Patients will be recruited consecutively until sample size of 150 is reached. This sample size is based on a priori power analysis using G*Power software. A sample size of 150 implies a power of 92% to detect a medium effect size (Cohen's f^2=0.15) using nine numerator degrees of freedom at a 0.05 level of significance.[26] Eligible patients will be adults (≥ 18 years), capable of providing informed consent and completing the survey in English or Kiswahili, who have a confirmed diagnosis of cancer in any tumor site within the past 12 months.

Surveys will include instruments related to demographics, initial symptoms experienced before cancer diagnosis, stigma, emotional well-being, and time from onset of symptoms and seeking medical help. The survey will conclude with 3-5 open-ended qualitative questions to discuss experiences of cancer-related stigma. In data analysis, we will measure the impact of cancer-related stigma and personal demographics as predictors of delays in screening, challenges in care engagement, and patient outcomes. Patients will receive 5000 Tanzanian shillings ($2.25) for participating. Survey data will be collected on study tablets and entered directly onto a secure electronic platform (REDCap), ideal for secure mobile offline data collection.

Objective 4: Develop the telehealth intervention with support from a study advisory board in Tanzania (Late 2022, Early 2023). I will form an advisory board consisting of PLWH (n=5), nurses (n=5), mental health workers (n=3), administrators and local and regional government officials (n=3), to assist in developing and refining the intervention. Advisory board members will be identified and invited to participate during in-depth interviews conducted for Objective 1. The advisory board will have approximately 16 members and will convene 3 times: 1) at the conclusion of Aim 1 to assist in refining the intervention prior to piloting by interacting with a prototype model, 2) midway through the clinical trial to review progress, and 3) at the conclusion of the trial to review and assist in interpreting findings. Key members of our previous KCMC advisory boards would be invited to continue their involvement for the current study.[27,28]

Employing principles of user-centered design[29,30] and an experimental therapeutic approach[31], we will invite advisory board members to experience the intervention as a study

participant would, by interacting with the intervention technology, counselors, and study surveys. The process will include eliciting continual, iterative feedback on the hypothesized mechanisms of change, to adapt the intervention model to the study-specific context.[29] Board members will observe as a mock patient completes screening and informed consent procedures, then uses a mobile phone to connect with an off-site counselor to participate in a mock counseling session. The board will give feedback on the process and perceived effectiveness of the session, as well as potential drawbacks. Through this process, we will reshape the intervention in real time, identifying opportunities to improve the intervention and increase potential acceptability for both clinic staff and patients.

Using advisory board feedback, the study team will develop a detailed study protocol and intervention manual. Counselors will be psychiatric nurses with an understanding of mental health and counseling skills. Counselor training will be provided by Co-I Dr. Knettel (a licensed psychologist) and the consulting psychiatrist Dr. Boshe, and will consist of 3 weeks of didactic and mock intervention sessions. This will include instruction in counseling skills, the intervention model, and ethical/safety considerations for research and counseling.

Objective 5: Pilot test the telehealth intervention in a randomized controlled trial to assess feasibility, acceptability, and potential efficacy for reducing suicidality and enhancing HIV care engagement. Preliminary Telehealth Model for Intervention Delivery (Timeline: 2023-2024). The proposed intervention will begin with implementing routine screening for emotional distress, including depression and suicidal ideation, in adult HIV care at the 3 study sites. The screening tools, the PHQ-2 and Columbia-Suicide Severity Rating Scale (C-SSRS), contain 8 items assessing depression and suicidality in the past month, including thoughts, intent, plan, and actions.[32,33]

Participants must be age 18 or older and capable of understanding and providing informed consent to participate. Patients will be eligible if they have been diagnosed with HIV and indicate actual thoughts of suicide in the past month. Participants will be excluded if they are under age 18 or physically or cognitively not capable of completing the study procedures.

Once consented, the study team will assist patients to make a video call to the telehealth hub using a mobile phone. Video calls will be made using WhatsApp, a freely available, secure, and widely used mobile application with end-to-end encryption. Calls will be made free of charge (without data charges) using Wi-Fi hotspots made available at each study clinic. Patients who do not have an Internet-equipped phone will use study-owned phones. Private space will be provided in the research offices at each clinic for video calls. The telehealth hub will be located in the KCMC Psychiatry Clinic and will be staffed by two mental health nurses. The two study nurses will answer calls using study-provided tablets and will document patient care electronically. The telehealth model will allow for the delivery of all sessions by nurse-counselors at the central hub to efficiently reach patients at subsidiary regional HIV clinics.

Sessions will be 30 minutes in length and held every two weeks to mirror the frequency of HIV clinic appointments for newly diagnosed patients. Additional support for high-acuity patients will be offered through weekly text message, phone contacts, and booster sessions. Session 1 will focus on safety planning, Session 2 will be guided by the HIV Stigma Framework, targeting stigma and misinformation as key contributors to emotional distress among PLWH.[12] In Session 3, the focus shifts to HIV care engagement and

medication adherence using CBT-AD. At the end of each session, the counselor will re-administer the C-SSRS and patients at acute risk will be accompanied to a mental health provider for further assessment.

To evaluate the intervention, we will enroll 60 PLWH in a pilot feasibility trial. After providing consent, participants will complete a baseline survey administered by trained study staff. Participants will receive an incentive of 10,000 TZ shillings ($4.50) at each contact to compensate for time and transport. Data collected at baseline will include measures of suicidality, HIV care engagement, and covariates such as stigma, social support, and quality of life. Upon completion of the baseline survey, participants will be individually randomized to receive either a brief Safety Planning Intervention (n=30), or the 3-session telehealth intervention (n=30).

After randomization, participants will be guided to a private space and assisted to connect by video call to the nurse-counselor (based at KCMC). Upon connecting, control participants will receive the brief (10-15 minute) Safety Planning Intervention and intervention participants will complete the first of 3 telehealth-delivered counseling sessions. Participants in both conditions will also have access to walk-in supportive counseling sessions at the study clinics throughout their participation in the study. These ‘booster sessions’ will be focused on assessing risk, safety planning, and reinforcing prior session content.

All participants will be called 3 months post-enrollment to return for a follow-up survey assessing study outcomes. Intervention participants will also complete measures of satisfaction with the intervention, barriers and facilitators to participation, and feedback for refinement. At the 3-month time point, we will also conduct a review of medical records to gather information on clinic-conducted viral load tests and corroborate self-reported clinic attendance during the study period. Half of the intervention participants (n=15) will also complete in-depth interviews (IDIs) to provide detailed feedback on their experiences of the intervention, its impact, and potential barriers to future implementation.

Figure 1. Clinical trial design and participant flow

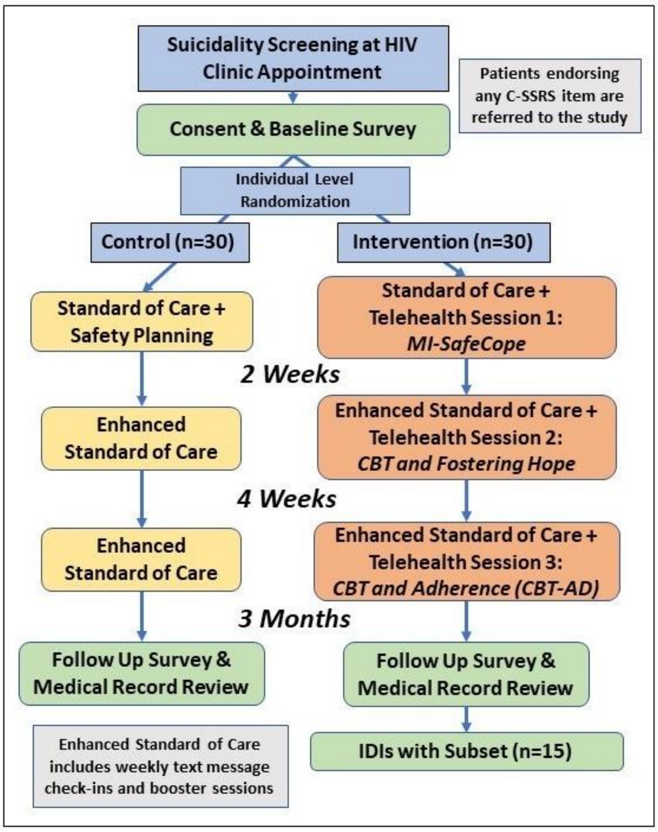

Data analysis. Feasibility and acceptability of the intervention will be described by retention patterns, participant satisfaction, fidelity, and implementation cost. Participant satisfaction will be measured using the Client Satisfaction Questionnaire[34] at the 3-month survey and defined as a mean score ≥ 3 on a 1-4 scale. Fidelity to the manual will be measured by TQS and deemed acceptable if mean scores are ≥ 3 on a 0-4 scale.

Potential efficacy will be assessed by analyzing differences by condition in primary outcomes (suicidality, HIV care engagement, viral load) and secondary outcomes (depression, social support, quality of life). Mixed-effects regression will be used to model pre-post differences within and between arms using a time by condition model specification (time, condition, and time*condition). Individual random intercepts will be used to account for correlation from repeated measurement.

Limitations:

Participants will be recruited from a small number of urban clinics in northern Tanzania. Thus, findings may not be generalizable to other settings, including more rural areas or other regions of the country. The services offered at the study clinics reflects the broader national healthcare strategy, but other clinics may have implemented other measures to improve services, leading to different patient outcomes. Additionally, rural clinics may lack some of the equipment and resources available in urban areas. Several of our study procedures will rely on participant self-report. We will attempted to corroborate self-reported variables using patient medical records and biomarkers (e.g., CD4 and viral load data), but these biomarkers may be unavailable for some participants.

Risks and Benefits:

The well-being of study participants is of utmost importance. The study procedures that have been selected are minimally invasive and associated with minimal risk; alternative procedures with lower risk are not available. Nevertheless, the protocol raises two general areas of human subjects concerns. First is confidentiality, particularly but not exclusively related to participants' HIV or cancer diagnoses, which is described in the next section. The second is the potential for emotional distress during the assessment. It is possible that in the patient interviews, participants may experience discomfort or embarrassment related to answering questions about their HIV history and pregnancy status. Participants will be informed prior to the assessment that they may choose to skip any question or procedure they find uncomfortable. The informed consent procedure will clearly notify participants of the potential risks of participation.

All research staff will be extensively trained on study procedures related to maintaining participant confidentiality and managing emotional distress. Staff will receive additional guidance on the conduct of interviews that elicit personal information, procedures for maintaining confidentiality, particularly related to participant's HIV status or cancer diagnosis, and the importance of being sensitive to and respectful of all participants. Research staff will also be trained on how to identify and manage participant distress, and when to terminate an interview and/or provide referrals for services.

Research staff will be knowledgeable about relevant social and health services and will be capable of accessing appropriate resources at KCMC. Staff will be trained by a U.S.-trained Licensed Psychologist on how to provide crisis management and make a personalized plan for safety in the event of extreme emotional distress or suicidal ideation with a plan and intent to act. If such a case were to arise, the staff member would stabilize the patient while making contact with the consulting psychiatrist (Dr. Boshe), who will help to facilitate transfer to the psychiatric nursing service at KCMC. Study procedures have been designed to minimize risk associated with the emotional content being addressed. Any acute risk will be documented via a log which will be kept in a secure, locked cabinet at the clinic, and research staff will follow up with these participants regarding their level of distress during subsequent research contacts.

The risk/benefit ratio for this study is relatively low, primarily because all of the data collection procedures are minimally invasive. All procedures will be performed by trained research staff to minimize risks, discomfort, and adverse events. We believe that the potential risks of this study, which are minimal and unlikely to occur, are reasonable given the potential benefits to inform future service implementation. This study poses no immediate physical risk to participants, and we have developed a thorough plan of action in the instance that one of the aforementioned adverse events occurs. Our study team has significant experience working with this population, and we are confident in both our assessment of potential risks and our ability to handle such risks. As such, we believe that the potential benefits outweigh the risks posed to human subjects. This research is expected to yield important new information on the implementation of the counseling intervention in Tanzania and similar settings.

Confidentiality:

Confidentiality is of critical importance, and we will take many precautions to protect against the possibility of a breach of confidentiality. Disclosure of medical or other personal information, particularly related to mental health, may pose personal or social risks. Our research team is very aware of the importance of maintaining strict confidentiality and has extensive experience dealing with sensitive information.

Procedures to maintain confidentiality

The following precautions will protect the privacy of participants and maintain confidentiality of research data: (1) All staff will be well trained in confidentiality and data security procedures. (2) Privacy will be maintained by conducting all study assessments and counseling sessions in closed and private rooms. At the time of enrollment, we will request participants give their preferences on how to be contacted, including their approval for text message and phone contacts. If the participant agrees to text message and phone contacts, these messages will not contain reference to HIV, cancer, or suicidal ideation. Instead, we will use generic language to maintain participant confidentiality, such as, "Hello,

this is the nurse from KCMC checking back about our discussion this week. Are there any follow-up topics you would like to discuss?" (3) Each participant will be assigned a unique study ID number, and all data will be de-identified and coded with ID numbers only. The key linking participant names and ID numbers will be stored in a separate password protected document on a password protected computer, to which only essential study staff will have access (i.e., principal investigators, study coordinator, and data analyst). (4) Data (including audio-recordings and qualitative transcripts) will be securely stored in locked file cabinets in locked offices and in password protected documents on password protected computers and secure servers. Access to data storage areas and computers will be restricted to the principal investigators, study coordinator, and data analyst. (5) Analysis will occur exclusively on de-identified data. (6) Data will only be stored for as long as necessary to complete the study, and for adherence to IRB regulations. Thus, while we acknowledge that a breach of confidentiality is possible, the likelihood is very low.

Informed consent:
Prior to participating in the study, participants will provide informed consent. A research assistant will describe the study in detail to the participant, allow him/her ample time to read the consent form thoroughly (or have it read to them) and ask questions, and ensure that he/she understands the purpose of the study, study procedures involved, and potential risks. Verification of comprehension of informed consent will be accomplished by asking participants to recall central points in the consent process. This procedure will also provide an opportunity to clarify any points of confusion. Participants will sign one copy of the informed consent form and will be given a full copy for their personal records, as well as a condensed version summarizing key points in non-technical terms. If the participant is not able to write his/her name, a thumb print can be used in lieu of a signature. Participants will be told at enrollment that their decision to participate or not will in no way affect their current or future employment or health care at the facility.

Dissemination of Results:

This intervention will seek to build local capacity for mental health support in HIV and cancer care, increase the quality of the services provided through a more theory-driven approach, and enhance HIV and cancer care engagement among patients served at the study sites. We believe that addressing the emotional well-being of patients will have a direct, positive impact on patient treatment outcomes and long-term health.

The Regional Medical Officer and clinic staff have indicated their support for this project and future training efforts that may arise from this research, detailing that the findings may serve to improve tratment quality at the study clinics and at other sites throughout the Kilimanjaro Region. Our research team has a long-standing relationship with the administrators, providers, and nurses at the study clinics, and therefore will partner closely with those individuals to carry out the research and implement findings to improve care for patients.

We will disseminate the findings of the study, first locally and then at larger venues nationally and internationally. This will include presentations at staff meetings in the clinics where the research will be conducted, describing the findings of the results, obtaining staff perspectives to assist in interpreting the results, and discussing opportunities to leverage the results to change clinic practices and improve patient care. We will then write these findings into research abstracts manuscripts with the assistance and input of clinic staff, who will be included as co-authors. Abstracts and manuscripts will be submitted to research

conferences and high-quality academic journals to maximize visibility and add to the literature on HIV and cancer stigma in sub-Saharan Africa and beyond.

Upon completion of this study, we expect to have the experience and data to support an application for a larger National Institute of Health (NIH) R01 grant to further refine and test the intervention model. This will include efforts to expand the intervention to other sites across Tanzania and work toward broader scale-up. Finally, we will also have an established local study team with the capacity to support future grants. We will emphasize the value of professional development for our Tanzanian collaborators, including supporting their academic and professional pursuits, advancing their research skills, and facilitating future opportunities in clinical research.

References

1. UNAIDS. *Country Fact Sheet: United Republic of Tanzania*. UNAIDS; 2019. https://www.unaids.org/en/regionscountries/countries/unitedrepublicoftanzania

2. The Cancer Atlas. Sub-Saharan Africa. The Cancer Atlas. Published 2021. Accessed March 11, 2021. http://canceratlas.cancer.org/n6X

3. Lyimo EP, Rumisha SF, Mremi IR, et al. Cancer Mortality Patterns in Tanzania: A Retrospective Hospital-Based Study, 2006-2015. *JCO Glob Oncol*. 2020;6:224-232. doi:10.1200/JGO.19.00270

4. World Health Organization. Shortages and late diagnosis hamper cancer treatment in Dar es Salaam. WHO. Published 2016. Accessed March 16, 2021. http://www.who.int/medicines/about/country-stories/tanzania-cancer-treatment/en/

5. Adatia A. Cancer in My Community: The Barriers to Cancer Care in Tanzania. American Society of Clinical Oncology. Published 2020. Accessed March 16, 2021. https://www.cancer.net/blog/2020-04/cancer-my-community-barriers-cancer-care-tanzania

6. Runge AS, Bernstein ME, Lucas AN, Tewari KS. Cervical cancer in Tanzania: A systematic review of current challenges in six domains. *Gynecologic Oncology Reports*. 2019;29:40-47. doi:10.1016/j.gore.2019.05.008

7. Mayston R, Kinyanda E, Chishinga N, Prince M, Patel V. Mental disorder and the outcome of HIV/AIDS in low-income and middle-income countries: a systematic review. *AIDS*. 2012;26:S117-S135. doi:10.1097/QAD.0b013e32835bde0f

8. Yehia BR, Stephens-Shield AJ, Momplaisir F, et al. Health Outcomes of HIV-Infected People with Mental Illness. *AIDS Behav*. 2015;19(8):1491-1500. doi:10.1007/s10461-015-1080-4

9. Chuah FLH, Haldane VE, Cervero-Liceras F, et al. Interventions and approaches to integrating HIV and mental health services: a systematic review. *Health Policy Plan*. Published online 2017. doi:10.1093/heapol/czw169

10. Safren SA, Bedoya CA, O'Cleirigh C, et al. Cognitive behavioural therapy for adherence and depression in patients with HIV: a three-arm randomised controlled trial. *Lancet HIV*. 2016;3(11):e529-e538. doi:10.1016/S2352-3018(16)30053-4

11. Abas M, Nyamayaro P, Bere T, et al. Feasibility and Acceptability of a Task-Shifted Intervention to Enhance Adherence to HIV Medication and Improve Depression in People Living with HIV in Zimbabwe, a Low Income Country in Sub-Saharan Africa. *AIDS Behav*. 2018;22(1):86-101. doi:10.1007/s10461-016-1659-4

12. Earnshaw VA, Smith LR, Chaudoir SR, Amico KR, Copenhaver MM. HIV Stigma Mechanisms and Well-Being among PLWH: A Test of the HIV Stigma Framework. *AIDS Behav*. 2013;17(5):1785-1795. doi:10.1007/s10461-013-0437-9

13. Watt MH, Minja L, Knettel BA, et al. Pilot outcomes of Maisha: An HIV stigma reduction intervention developed for antenatal care in Tanzania. *AIDS and behavior*. Published online 2020.

14. Chan BT, Tsai AC, Siedner MJ. HIV Treatment Scale-Up and HIV-Related Stigma in Sub-Saharan Africa: A Longitudinal Cross-Country Analysis. *Am J Public Health*. 2015;105(8):1581-1587. doi:10.2105/AJPH.2015.302716

15. Meacham E, Orem J, Nakigudde G, Zujewski JA, Rao D. Exploring stigma as a barrier to cancer service engagement with breast cancer survivors in Kampala, Uganda. *Psychooncology*. 2016;25(10):1206-1211. doi:10.1002/pon.4215

16. Oystacher T, Blasco D, He E, et al. Understanding stigma as a barrier to accessing cancer treatment in South Africa: implications for public health campaigns. *Pan Afr Med J*. 2018;29:73. doi:10.11604/pamj.2018.29.73.14399

17. Rosser JI, Njoroge B, Huchko MJ. Cervical cancer stigma in rural Kenya: What does HIV have to do with it? *J Cancer Educ*. 2016;31(2):413-418. doi:10.1007/s13187-015-0843-y

18. Wallace M, Bos A, Noble C. Cancer-Related Stigma in South Africa: Exploring Beliefs and Experiences Among Cancer Patients and the General Public. *JGO*. 2018;4(Supplement 2):112s-112s. doi:10.1200/jgo.18.53700

19. Knettel BA, Rugira J, Tesha F. “They will start believing in counseling”: Provider perceptions of the presentation and treatment of mental illness in northern Tanzania. *International Perspectives in Psychology: Research, Practice, Consultation*. 2018;7(1):4-18. doi:10.1037/ipp0000078

20. Andersen LS, Magidson JF, O’Cleirigh C, et al. A pilot study of a nurse-delivered cognitive behavioral therapy intervention (Ziphamandla) for adherence and depression in HIV in South Africa. *J Health Psychol*. 2018;23(6):776-787. doi:10.1177/1359105316643375

21. Czyz EK, King CA, Biermann BJ. Motivational Interviewing-Enhanced Safety Planning for Adolescents at High Suicide Risk: A Pilot Randomized Controlled Trial. *Journal of Clinical Child & Adolescent Psychology*. 2019;48(2):250-262. doi:10.1080/15374416.2018.1496442

22. TNBS. *Tanzania Total Population by District-Regions: 2016-2017*. Tanzania National Bureau of Statistics; 2017. Accessed November 15, 2019. https://www.nbs.go.tz/nbs/takwimu/census2012/Tanzania_Total_Population_by_District-Regions-2016_2017r.pdf

23. Charmaz K. *Constructing Grounded Theory*. 2nd edition. Sage; 2014.

24. Guest G, MacQueen KM, Namey EE. *Applied Thematic Analysis*. SAGE; 2012.

25. Campbell JL, Quincy C, Osserman J, Pedersen OK. Coding In-depth Semistructured Interviews: Problems of Unitization and Intercoder Reliability and Agreement. *Sociological Methods & Research*. 2013;42(3):294-320. doi:10.1177/0049124113500475

26. Carter-Harris L, Hermann CP, Schreiber J, Weaver MT, Rawl SM. Lung cancer stigma predicts timing of medical help-seeking behavior. *Oncol Nurs Forum*. 2014;41(3):E203-210. doi:10.1188/14.ONF.E203-E210

27. Watt MH, Knippler ET, Minja L, et al. A counseling intervention to address HIV stigma at entry into antenatal care in Tanzania (Maisha): study protocol for a pilot randomized controlled trial. *Trials*. 2019;20(1):807. doi:10.1186/s13063-019-3933-z

28. Watt MH, Knettel BA, Knippler ET, et al. The development of Maisha, a video-assisted counseling intervention to address HIV stigma at entry into antenatal care in Tanzania. *Evaluation and Program Planning*. 2020;83:101859. doi:10.1016/j.evalprogplan.2020.101859

29. Lyon AR, Koerner K. User-Centered Design for Psychosocial Intervention Development and Implementation. *Clin Psychol (New York)*. 2016;23(2):180-200. doi:10.1111/cpsp.12154

30. Harte R, Glynn L, Rodríguez-Molinero A, et al. A Human-Centered Design Methodology to Enhance the Usability, Human Factors, and User Experience of Connected Health Systems: A Three-Phase Methodology. *JMIR Hum Factors*. 2017;4(1):e8. doi:10.2196/humanfactors.5443

31. NIMH. Support for Clinical Trials at NIMH. Accessed July 8, 2020. https://www.nimh.nih.gov/funding/opportunities-announcements/clinical-trials-foas/index.shtml

32. Kroenke K, Spitzer RL, Williams JBW. The Patient Health Questionnaire-2: Validity of a Two-Item Depression Screener. *Medical Care*. 2003;41(11):1284-1292. doi:10.1097/01.MLR.0000093487.78664.3C

33. Posner K, Brown GK, Stanley B, et al. The Columbia-Suicide Severity Rating Scale: initial validity and internal consistency findings from three multisite studies with adolescents and adults. *Am J Psychiatry*. 2011;168(12):1266-1277. doi:10.1176/appi.ajp.2011.10111704

34. Attkisson CC, Greenfield TK. The UCSF Client Satisfaction Scales: I. The Client Satisfaction Questionnaire-8. In: *The Use of Psychological Testing for Treatment Planning and Outcomes Assessment, 2nd Ed*. Lawrence Erlbaum Associates Publishers; 1999:1333-1346.
