# Supplementary material for: Protocol for a pilot randomized controlled trial of a telehealth-delivered counseling intervention to reduce suicidality and improve HIV care engagement in Tanzania
Source: PLoS One. 2023 Jul 27;18(7):e0289119. doi: 10.1371/journal.pone.0289119 (PMC10374000; doi:10.1371/journal.pone.0289119)
Supplement: S1 Appendix — (PDF) [file pone.0289119.s003.pdf]

Clinic Name: Date:

Nurse Name:

Fikra Zenye Matumaini – Nurse Screening Tool

Description: Please listen closely and tell me how often you have been bothered by the following problems over the past 2 weeks.

Tafadhali sikiliza kauli zifuatazo na uniambie ni kwa kiasi gani umesumbuliwa na kila moja ya matatizo haya kwa kipindi cha siku 14 (wiki mbili) zilizopita.

	**Not at all** Hapana kabisa (siku 0)	**Several days** Siku Kadhaa (1-7)	**More than half the days** Zaidi ya siku 7	**Nearly every day** Karibu kila siku ndani ya siku 14
1. Little interest or pleasure in doing things<br>1. Hamu au raha kidogo ya kufanya vitu	0	1	2	3
2. Feeling down, depressed, or hopeless<br>2. Kujisikia kuwa na huzuni, kusononeka au kukosa matumaini	0	1	2	3

Combined score for Items 1 and 2: _______________
Jumla ya alama kwa Swali la 1 na 2: _______________

3. In the last month, have you had any actual thoughts of killing yourself?
3. Kwa kipindi cha mwezi moja uliopita, umewahi kupata mawazo ya kujiua?

YES NO
NDIO HAPANA

Screener Outcomes:

See the combined score for Items 1 and 2. Is the score 3 or higher? **YES NO**
Angalia jumla ya alama kwa Swali la 1 na 2. Je, ni 3 au zaidi? **NDIO HAPANA**
Did the patient respond YES on item 3? **YES NO**
Je, mteja amejibu "NDIO" kwa swali la 3? **NDIO HAPANA**

If you answered YES to either of these two items, please contact the Fikra Zenye Matumaini team (include name and contact info here).
Kama umejibu "NDIO" kwenye moja kati ya maswali haya, tafadhali wasiliana na timu ya FIKRA ZENYE MATUMAINI.

For Study Team Use Only:
Was this patient eligible for brief support for depression? **YES NO**
Was this patient eligible for the Fikra Zenye Matumaini study? **YES NO**
Was this patient referred to the team? **YES NO**
If referred, give study ID:

If eligible but not referred, give reason and how you followed up:
