# Supplementary material for: Protocol for a pilot randomized controlled trial of a telehealth-delivered counseling intervention to reduce suicidality and improve HIV care engagement in Tanzania
Source: PLoS One. 2023 Jul 27;18(7):e0289119. doi: 10.1371/journal.pone.0289119 (PMC10374000; doi:10.1371/journal.pone.0289119)
Supplement: S2 Appendix — (PDF) [file pone.0289119.s004.pdf]

Study ID ___________________ **Date of Enrollment** _______________

Participant Name __________________________________

Participant Primary Phone Number ____

Participant Alternative Phone Numbers ____

____

Alternative Contact(s) that can be contacted if participant cannot be reached
(Note: these contacts can also be contacted in case in emergency) Include name/relation, contact number, what information the RA can mention

____

____

____

Physical address
If we can't reach you by phone, is it okay to reach out to you in person? What is the easiest way to find you?

____

____

Communication preferences (time of day, etc.)

____

DATE of session 2 ___________________ scheduled Session of session ___________________________
