# Supplementary material for: Protocol for a pilot randomized controlled trial of a telehealth-delivered counseling intervention to reduce suicidality and improve HIV care engagement in Tanzania
Source: PLoS One. 2023 Jul 27;18(7):e0289119. doi: 10.1371/journal.pone.0289119 (PMC10374000; doi:10.1371/journal.pone.0289119)
Supplement: S3 Appendix — (PDF) [file pone.0289119.s005.pdf]

Quantitative Survey – Baseline & 3m

0. Cover Page		
TIMEPOINT	Timepoint	1. Baseline<br>2. 3 Months
INTERVIEWER	Interviewer	
CLINIC	Clinic	1. Majengo<br>2. Mawenzi<br>Other _____________
STUDY_ID	Study ID	_______________
3M SURVEY ONLY: GROUP	What condition was the participant randomized to?	1. Comparison<br>2. Intervention
SURVEY_DATE	Date of Survey	D/M/Y

DEMOGRAPHICS SECTION ASKED AT BASELINE ONLY

1. Demographics (DEM)		
DEM1	How old are you?<br>Una miaka mingapi?	
DEM2	What is your sex/gender?<br>Jinsia yako ni ipi?	1. Female/ Mwanamke<br>2. Male/Mwanaume
DEM3	How far do you live from this clinic (in kilometers)?<br>Mahali unapoishi ni mbali kwa kiasi gani kutoka hapa kliniki (Kwa kilometa)?	

DEM4	What is your religion?<br>Wewe ni wa dini gani?	1. Christian, denomination: Mkristo, madhehebu:<br>______________________<br>2. Muslim Mwislamu<br>3. No religion Sina dini<br>4. Other religion: Dini nyingine:<br>_________________________
DEM5	What is the highest level of school that you completed?<br>Ni kiwango gani cha juu cha elimu umekifikia?	0. *No formal education Sina Elimu*<br>1. *Standard 1 Darasa la kwanza*<br>2. *Standard 2 Darasa la pili*<br>3. *Standard 3 Darasa la tatu*<br>4. *Standard 4 Darasa la nne*<br>5. *Standard 5 Darasa la tano*<br>6. *Standard 6 Darasa la sita*<br>7. *Standard 7 Darasa la saba*<br>8. *Secondary education* (*Specify level attained*: *Elimu ya sekondari (elezea kiwango ulichofikia)*:_______________<br>9. *Higher education* (*Specify field*: *Elimu ya juu (elezea kiwango ulichofikia*:____________________
DEM6	What best describes your current relationship status?<br><br>Nini kinaelezea vizuri zaidi hali yako ya mahusiano kwa sasa?	1. Married Nimeolewa/Nimeoa<br>2. Single, not in a relationship Sijaolewa/Sijaoa, siko kwenye mahusiano<br>3. In a relationship, but not married Niko kwenye mahusiano, ila sijaolewa/sijaoa<br>4. Separated from spouse/ Divorced Nimetengana na mume au mke wangu/ Mtalaka<br>5. Widow Mjane/Mgane

If married or in a relationship CONTINUE. If not SKIP to **SES 1**		
DEM7	Do you currently live with your partner?<br>Je, kwa sasa hivi unaishi na mwenza/mpenzi wako?	**1.** Yes, all the time<br>Ndiyo, wakati wote<br>**2.** Yes, sometimes<br>Ndiyo, wakati mwingine<br>**3.** No<br>Hapana
SES SECTION ASKED AT BASELINE ONLY<br>**2. Socio-Economic Status (SES)**		
SES1	Does the house where you live have electricity?<br>Nyumba unayoishi ina umeme?	0. No Ndiyo<br>1. Yes Hapana
SES2	Does the house where you live have piped water inside?<br>Nyumba unayoishi ina bomba la maji la ndani?	0. No Ndiyo<br>1. Yes Hapana
SES3	Do you have a television in your home?<br>Je, kuna televisheni nyumbani kwako?	0. No Ndiyo<br>1. Yes Hapana
SES4	Do you have a refrigerator in your home?<br>Je kuna friji nyumbani kwako?	0. No Ndiyo<br>1. Yes Hapana
SES5	Are you currently working?<br>Je unafanya kazi kwa sasa?	0. No Ndiyo<br>1. Yes Hapana
SES6	***If working:*** What type of work do you do?<br>***Kama ana kazi:*** Unafanya kazi ya aina gani?	
SES7	In total, what is the combined monthly income in your household? (give best estimate)<br>Kwa ujumla, kipato cha kaya kwa mwezi? Ukijumlisha vipato vya wakaazi wote kwenye kaya yako (toa makadirio bora)	___________________TSh

HFIAS SECTION ASKED AT BASELINE ONLY<br>**3. Modified Household Food Insecurity Access Scale (HFIAS) and Social Determinants of Health (SDH)**		
**Description:** I am now going to ask you some questions about your household, and how easy or difficult it is to get food and other resources you need. Your responses in this section should reflect yourself and the people who live with you and who you feel financially responsible for.<br><br>**Maelezo:** Sasa nitakuuliza maswali machache kuhusu nyumbani kwako, ni rahisi au vigumu kiasi gani kupata chakula na mahitaji mengine unayohitaji. Majibu yako katika kipengele hiki yanapaswa kukulenga wewe na watu ambao unaoishi nao na wanaokutegemea kifedha.		
HFIAS1	In the past month, how often could you not feed your family?<br>Katika mwezi uliopita, ni mara ngapi hukuweza kuilisha familia yako?	0. Never Hakuna<br>1. Once or twice Mara moja au mbili<br>2. More than once or twice but not every day Zaidi ya mara moja au mbili lakini si kila siku<br>3. Every day Kila siku
HFIAS2	In the past month, how often were you hungry, but did not eat because you could not afford enough food?<br>Katika mwezi uliopita, ni mara ngapi ulikuwa na njaa, lakini hukula kwa sababu hukuweza kununua chakula cha kutosha?	0. Never Hakuna<br>1. Once or twice Mara moja au mbili<br>2. More than once or twice but not every day Zaidi ya mara moja au mbili lakini si kila siku<br>3. Every day Kila siku
HFIAS3	In the past month, how often did you or other adults in your household not eat for a whole day because there was not enough money for food?<br>Katika mwezi uliopita, ni mara ngapi wewe au mtu mzima mwingine katika kaya yako hakuweza kula kwa siku nzima kwa sababu hapakuwa na pesa za kutosha kwa ajili ya chakula?	0. Never Hakuna<br>1. Once or twice Mara moja au mbili<br>2. More than once or twice but not every day Zaidi ya mara moja au mbili lakini si kila siku<br>3. Every day Kila siku
HFIAS4	In the past month, how often did you or other adults in your household cut the	0. Never Hakuna<br>1. Once or twice Mara moja au mbili

	size of your meals because there was not enough money for food?<br>Katika mwezi uliopita, ni mara ngapi wewe au mtu mzima wa kaya yako alipunguza kiasi cha chakula chenu kwa siku nzima kwa sababu hapakuwa na pesa za kutosha kwa ajili ya chakula?	2. More than once or twice but not every day Zaidi ya mara moja au mbili lakini si kila siku<br><br>3. Every day Kila siku
SDH1	In the past month, how often did you worry that you or your family members didn't have a safe, stable place to sleep and store your possessions?<br>Katika mwezi uliopita, ni mara ngapi ulikuwa na wasiwasi kwamba wewe au mtu wa nyumbani kwako hamkuwa na mahali salama pa kulala na kuhifadhi mali zenu?	0. Never Hakuna<br><br>1. Once or twice Mara moja au mbili<br><br>2. More than once or twice but not every day Zaidi ya mara moja au mbili lakini si kila siku<br><br>3. Every day Kila siku
SDH2	In the past month, how often did you have challenges clothing yourself and your family (including normal daily clothing, school or work uniforms)?<br>Katika mwezi uliopita, ni mara ngapi ulikuwa na changamoto za kujihudumia kimavazi wewe na familia yako? (ni pamoja na mavazi ya kawaida ya kila siku, sare za shule au sare za kazini)	0. Never Hakuna<br><br>1. Once or twice Mara moja au mbili<br><br>2. More than once or twice but not every day Zaidi ya mara moja au mbili lakini si kila siku<br><br>3. Every day Kila siku
SDH3	In the past month, how often were you worried about having fare for transport to places you needed to go?<br>Katika mwezi uliopita, ni mara ngapi ulikuwa na wasiwasi kuhusu kuwa na nauli ya usafiri kwenda maeneo unayohitaji kwenda?	0. Never Hakuna<br><br>1. Once or twice Mara moja au mbili<br><br>2. More than once or twice but not every day Zaidi ya mara moja au mbili lakini si kila siku<br><br>3. Every day Kila siku
SDH4	In the past month, how often were you worried about debts, such as money owed by you or owed by someone in your household?<br>Katika mwezi uliopita, ni mara ngapi ulikuwa na wasiwasi kuhusu madeni,	0. Never Hakuna<br><br>1. Once or twice Mara moja au mbili<br><br>2. More than once or twice but not every day Zaidi ya mara moja au mbili lakini si kila siku

	kama vile pesa unazodaiwa au anazodaiwa mtu wa nyumbani kwako?	3. Every day Kila siku
SDH5	In the past month, how often were you worried about not having capital for your business or income-generating activities? Katika mwezi uliopita, ni mara ngapi ulikuwa na wasiwasi kuhusu kutokuwa na mtaji wa biashara yako au shughuli za kukuingizia kipato?	0. Never Hakuna<br>1. Once or twice Mara moja au mbili<br>2. More than once or twice but not every day Zaidi ya mara moja au mbili lakini si kila siku<br>3. Every day Kila siku

4. HIV History (HIV)

HIV1	BASELINE ONLY<br>When did you first learn that you were HIV positive?<br>Ni wakati gani uligundua kwa mara ya kwanza kwamba una VVU?	______ days ago<br>Siku ______ zilizopita<br>______ months ago<br>Miezi ______ iliyopita<br>______ years ago<br>Miaka ______ iliyopita<br>(Be as specific as possible/ jitahidi kuainisha kwa ufasaha)<br>☐ Check here if born with HIV; also include # of years above<br>☐ Tiki hapa ikiwa kama alizaliwa na VVU; pia andika na miaka hapa juu
HIV2	BASELINE ONLY<br>**If in a relationship:** What is your partner's HIV status?<br>**Ikiwa yuko katika uhusiano:** Je, mwenzako ana hali gani ya VVU?	0. HIV negative<br>1. HIV positive<br>2. Don't know or hasn't been tested<br>3. Don't have a partner
HIV7_F	3 MONTH ONLY<br>At any point since you enrolled in the study, have you switched clinics for your HIV care?	0. No<br>1. Yes (Describe):

	Tangu ulipoingia kwenye mradi wa utafiti, je umewahi kuhama/kubadili kituo chako cha huduma ya VVU?	
For this section, ask to see the patient's HIV clinic card.		
HIV4	BASELINE ONLY<br>What ARV regimen are you currently using?<br>Ni mchanganyiko gani wa dawa za kurefusha maisha (ARV) unaoutumia kwa sasa?<br>(***check patient card or pill bottle***)	1. *Dolutegravir*<br>2. *TLE (Tenofovir, Lamivudine and Efavirenz)*<br>3. *Other:* _______________<br>8. *Information not available*
HIV5	BASELINE ONLY<br>When did you start taking ARVs?<br>Ulianza lini kutumia dawa za kurefusha maisha (ARV)? (***check card, or give patient's best estimate***)	_____ / _____ / _____<br>dd mm yy<br>siku mwezi mwaka<br>8. *Information not available*
HIV6	BASELINE ONLY<br>Since you started taking ARVs, have you had any gaps where you were not taking them?<br>Tangu ulipoanza kutumia ARVs, umeshawahi kuwa na vipindi ambavyo ulikuwa unaacha kumeza dawa?	0. No Hapana<br>1. Yes Ndiyo. Describe Elezea:

5. Treatment Adherence Assessment (TAA)

Description: I am going to ask you some questions about the HIV antiretroviral medications that you were prescribed to treat your HIV.

Maelezo: Nitakuuliza maswali kuhusu dawa za kurefusha maisha ambazo uliandikiwa kutibu VVU.

TAA1	Have you ever been prescribed ARVs?<br>Je umeshawahi kupewa dawa za kupunguza makali ya VVU?	0. No<br>1. Yes
TAA2	Have you ever taken ARVs?<br>Umeshawahi kumeza dawa za kupunguza makali ya VVU?	0. No

		1. Yes
TAA3	Within the last 14 days, have you taken ARVs?<br>Katika kipindi cha siku 14 zilizopita, umewahi kumeza dawa za kupunguza makali ya VVU?	0. No<br>1. Yes
TAA4	Think about the last 14 days. On how many *days* did you miss taking your HIV pill(s)?<br>Katika siku 14 zilizopita ni *siku* ngapi ulikosa kumeza vidonge vyako vya VVU?	[***Write down days: 0-14***]
TAA5	Within the last 90 days, have you taken ARVs?<br>Katika kipindi cha siku 90 zilizopita, umewahi kumeza dawa za kupunguza makali ya VVU?	0. No<br>1. Yes
TAA6	Think about the last 90 days. On how many *days* did you miss taking your HIV pill(s)?<br>Katika siku 90 zilizopita ni *siku* ngapi ulikosa kumeza vidonge vyako vya VVU?	[***Write down days: 0-90***]

6. HIV Disclosure (DISC)

Description: I would now like to hear about your experience talking to people in your life about your HIV status. If you have not talked to anyone about your status, or don't have plans to talk to anyone about your status, that is completely understandable and it is fine to say so. ADDITIONAL DESCRIPTION FOR 3-MONTH SURVEY: When we first met three months ago, you told me about the people you had disclosed to at that time. I would like to know about any new people you have disclosed to since then.

Maelezo: Sasa ningependa kusikia uzoefu wako wa kuongea na watu walioko maishani mwako kuhusu hali yako ya VVU. Kama hujaongea na mtu yeyote kuhusu hali yako, au huna mpango wa kuongea na mtu yeyote kuhusu hali yako, hiyo inaeleweka kabisa na ni sawa kusema hivyo. ADDITIONAL DESCRIPTION FOR 3-MONTH SURVEY: Tulipokutana kwa mara ya kwanza miezi mitatu iliyopita, uliniambia kuhusu watu uliowambia kuhusu hali yako ya maambukizi kwa wakati ule. Ningependa kujua kama kuna mtu mwingine yeyote ambaye umemwambia kuhusu hali yako ya maambukizi

		**No**<br>**Hapana**	**Yes**<br>**Ndiyo**
DISC1	BASELINE<br>Have you ever told another person about your HIV status?<br>Umewahi kumwambia mtu mwingine kuhusu hali yako ya VVU?	0<br>[***skip to PHQ1***]	1
DISC1_F	3-MONTH<br>Since enrolling in the study 3 months ago, have you told another person about your HIV status?	0<br>[***skip to PHQ1***]	1

	Tangu ulipojiandikisha katika utafiti miezi 3 iliyopita, umewahi kumwambia mtu mwingine kuhusu hali yako ya VVU?		
DISC2	BASELINE<br>***If in a relationship:*** Have you told your partner?<br>***Kama yupo kwenye mahusiano:*** Umemwambia mwenzi/mpenzi wako?	0	1
DISC2_F	3-MONTH<br>***If in a relationship:*** Since enrolling in the study 3 months ago, have you told your partner?<br>Tangu ulipojiandikisha katika utafiti miezi 3 iliyopita, umemwambia mwenzi/mpenzi wako?	0	1
DISC3	BASELINE<br>Have you told any family members?<br>Umewaambia ndugu yoyote katika familia?	0	1
DISC3b	BASELINE<br>***If yes:*** Which family members?<br>***Kama ndio:*** Ni mwanafamilia yupi?		
DISC3_F	3-MONTH<br>Since enrolling in the study 3 months ago, have you told any family members?<br>Tangu ulipojiandikisha katika utafiti miezi 3 iliyopita, umewaambia ndugu yoyote katika familia?	0	1
DISC3b_F	3-MONTH<br>***If yes:*** Which family members?<br>***Kama ndio:*** Ni mwanafamilia yupi?		
DISC4	BASELINE<br>Have you told any friends?<br>Umewaambia marafiki wowote?	0	1
DISC4_F	3-MONTH<br>Since enrolling in the study 3 months ago, have you told any friends?<br>Tangu ulipojiandikisha katika utafiti miezi 3 iliyopita, umewaambia marafiki wowote?	0	1
DISC5	BASELINE<br>Have you told anyone else?<br>Umemwambia mtu mwingine yeyote?	0	1
DISC5b	BASELINE<br>***If yes:*** Who?<br>***Kama ndio:*** Ni nani?		
DISC5_F	3-MONTH<br>Since enrolling in the study 3 months ago, have you told anyone else?<br>Tangu ulipojiandikisha katika utafiti miezi 3 iliyopita, umemwambia mtu mwingine yeyote?	0	1

DISC5b _F	3-MONTH<br>***If yes:*** Who?<br>***Kama ndio:*** Ni nani?	

At this point, I want to acknowledge that we are talking about topics that are quite personal. I want to reassure you that everything you share will remain confidential, we will not attach your name to any of your responses, and I hope you'll feel comfortable being open about these topics.

Sasa, ninapenda kutambua kwamba tunaongea kuhusu maswala ambayo ni ya binafsi/siri sana. Ninataka nikuhakikishie kwamba kila kitu utakachoniambia kitabaki kuwa siri, hatutaambatanisha jina lako kwenye majibu yako yoyote, na natumaini kuwa utajisikia huru kuongea kwa uwazi kuhusu maswala haya.

7. Patient Health Questionnaire (PHQ-9)

Description: For the following items, please listen closely and tell me how often you have been bothered by any of these problems over the past 2 weeks.

Maelezo: Tafadhali sikiliza kauli zifuatazo na uniambie ni kwa kiasi gani umesumbuliwa na kila moja ya matatizo haya kwa kipindi cha wiki 2 zilizopita.

		**Not at all** **Hapana kabisa**	**Several days** **siku kadhaa**	**More than half the days** **Zaidi ya siku 7**	**Nearly every day** **Karibu Kila siku**
PHQ1	Little interest or pleasure in doing things<br>Hamu au raha kidogo ya kufanya vitu	0	1	2	3
PHQ2	Feeling down, depressed, or hopeless<br>Kujisikia kuwa na uzuni, kusononeka au kukosa tumaini	0	1	2	3
PHQ3	Trouble falling asleep, staying asleep, or sleeping too much<br>Tatizo kupata usingizi au kuendelea kulala baada ya kupata usingizi au kulala sana	0	1	2	3
PHQ4	Feeling tired or having little energy<br>Kujisikia kuchoka au kuwa na nguvu kidogo	0	1	2	3
PHQ5	Poor appetite or overeating<br>Kutokuwa na hamu ya kula au kula kupita kiasi	0	1	2	3

PHQ6	Feeling bad about yourself – or that you're a failure or have let yourself or your family down<br>Kujisikia vibaya binafsi, au kusikia umeshindwa au umejishusha au umeangusha familia yako	0	1	2	3
PHQ7	Trouble concentrating on things, such as reading the newspaper or watching television<br>Kushindwa kutuliza akili katika vitu mfano kusoma gazeti au kuangalia runinga (TV)	0	1	2	3
PHQ8	Moving or speaking so slowly that other people could have noticed. Or, the opposite – being so fidgety or restless that you have been moving around a lot more than usual<br>Kutembea au kuongea polepole sana mpaka watu wengine wamegundua? Au kinyume chake kuwa na mashaka/wasiwasi au kutotulia kiasi hata umekuwa ukitembea tembea sana kuliko kawaida	0	1	2	3
PHQ9	Thoughts that you would be better off dead or of hurting yourself in some way<br>Fikra ya kwamba ni heri ukifa, au fikira za kujiumiza kwa njia fulani	0	1	2	3

PHQ10: ***If participant endorsed any of the above items:*** How difficult have those problems made it for you to do your work, take care of things at home, or get along with other people?

If participant endorsed any of the above items: Je, matatizo haya yamekuletea ugumu gani katika kufanya kazi, kushughulikia vitu nyumbani au kuelewana na watu wengine?

Not difficult at all<br>Hakuna ugumu kabisa	Somewhat difficult<br>Ngumu kiasi	Very difficult<br>Ngumu sana	Extremely difficult<br>Ngumu kuzidi

8. Columbia-Suicide Severity Rating Scale (C-SSRS) – Screen Version

Description: In this section, I am going to ask you some questions about how you have been feeling recently. Please answer these questions openly and honestly.

Maelezo: Kwenye kipengele hichi, nitakuuliza maswali kadhaa kuhusu ulivyokua unajisikia hivi karibuni. Tafadhali naomba unijibu maswali haya kwa ukweli na uwazi.

	Past month		Lifetime	
**(Ask Questions 1 and 2 to all participants)**	**NO**	**YES**	**NO**	**YES**
**1) Have you wished you were dead or wished you could go to sleep and not wake up?**<br>Uliwahi kutamani ungekufa au ulitamani ungelala na usiamke?<br>(CSSRS1_mo & CSSRS1_lt)				
**2) Have you had any actual thoughts of killing yourself?**<br>Umewahi kupata mawazo ya kujiua?<br>(CSSRS2_mo & CSSRS2_lt)				
**If YES to Question 2 (Lifetime), ask questions 3-7. If NO to 2, ask ONLY questions 6 and 7**	**Past month**		**Lifetime**	
	**NO**	**YES**	**NO**	**YES**
**3) Have you been thinking about how you might do this?**<br>Je, umekua ukiwaza utalifanyaje hili jambo?<br>(CSSRS3_mo & CSSRS3_lt)				
**4) Have you had these thoughts and had some intention of acting on them?**<br>Mark no for "I have the thoughts but I will not do anything about them."<br>Je, umekua ukipata haya mawazo na kuwa na nia ya kuyafanyia kazi?<br>(CSSRS4_mo & CSSRS4_lt)				
**5) Have you started to work out or worked out the details of how to kill yourself? Do you intend to carry out this plan?**<br>Je, umeanza kulifanyia kazi au kufanyia kazi jinsi utakavyojiua? Umedhamiria kutekeleza huu mpango?<br>(CSSRS5_mo & CSSRS5_lt)				

	Past 3 Months		Lifetime	
**6) Have you ever done anything, started to do anything, or prepared to do anything to end your life?**	**NO**	**YES**	**NO**	**YES**

Examples: Collected pills, obtained a gun, gave away valuables, wrote a will or suicide note, took out pills but didn't swallow any, held a gun but changed your mind or it was grabbed from your hand, went to the roof but didn't jump.<br>**Umeshawahi kufanya lolote, kuanza chochote au kujiandaa kufanya chochote kumaliza maisha yako?**<br>Mifano: kukusanya dawa, kupata bunduki, kugawa vitu vya thamani, kuandika wosia au barua ya kujiua, kuchukua madawa lakini haukumeza yoyote, kushika bunduki lakini ukabadili mawazo au ukapokonywa, umepanda darini/kwenye bati lakini haukuruka.<br>(CSSRS6_3mo & CSSRS6_lt)				
**7) Have you made a suicide attempt?**<br>Examples: Actually took pills or poison, tried to shoot yourself, cut yourself, tried to hang yourself, etc.<br>**Je umefanya jaribio la kujiua?**<br>Mifano: Kumeza madawa au sumu, kujaribu kujipiga risasi, kujikata, kujaribu kujinyonga nk.<br>(CSSRS7_3mo & CSSRS7_lt)				

Response Protocol to C-SSRS Screening:
If participant responds yes to ANY of these items in the past month or 3 months - 3, 4, 5, 6, or 7 – you must make an appropriate professional referral at the conclusion of the session.

Category 1 – Wish to be Dead<br>Category 2 – Non-specific Active Suicidal Thoughts<br>Category 3 – Active Suicidal Ideation without Intent to Act<br>Category 4 – Active Suicidal Ideation with Some Intent to Act<br>Category 5 – Active Suicidal Ideation with Specific Plan and Intent to Act<br>Category 6 – Preparatory Acts or Behavior<br>Category 7 – Aborted Attempt<br>Category 8 – Interrupted Attempt<br>Category 9 – Actual Attempt (non-fatal)<br>Category 10 – Completed Suicide	
C-SSRS_L: *Using the scale above, what is the highest category this patient has reached in his/her lifetime?*	*Category:*
C-SSRS_M: *Using the scale above, what is the highest category this patient has reached in the past month?*	*Category:*

9. Self-Efficacy to Avoid Suicidal Action (SEASA) Scale

Description: Please read each of the statements below carefully and circle the number which best fits how certain you are about how you would act in each of the following situations.

Maelezo: Tafadhali soma kila kauli zifuatazo kwa umakini na zungushia namba ambayo ni inakaribiana zaidi na uhakika ulionao wa jinsi ambavyo utafanya katika kila mazingira/hali zifuatazo.

		**Very Uncertain** **Sina Hakika kabisa** ... **Very Certain** **Nina Hakika Kabisa**
SEASA1	How confident are you that you WILL NOT attempt suicide in the future?<br>Una uhakika kiasi gani kwamba HAUTAJARIBU kujiua katika siku za mbeleni?	0 1 2 3 4 5 6 7 8 9 10
SEASA2	If you have serious thoughts of killing yourself in the future, how confident are you that you WILL BE ABLE to keep yourself from attempting suicide?<br>Kama una mawazo makali ya kutaka kujiua katika siku zijazo, una uhakika kiasi gani kuwa UTAWEZA kujizuia kujaribu kujiua?	0 1 2 3 4 5 6 7 8 9 10
SEASA3	If you have thoughts of killing yourself in the future, how confident are you that you WILL tell someone?<br>Kama una mawazo ya kutaka kujiua katika siku zijazo, una uhakika kiasi gani kwamba UTAMWAMBIA mtu mwingine?	0 1 2 3 4 5 6 7 8 9 10
SEASA4	How certain are you that you could control future thoughts of suicide if you were experiencing physical or emotional pain?<br>Kwa kiasi gani una uhakika kwamba utaweza kuyazuia mawazo ya kutaka kujiua ikiwa kama utakuwa unapitia kipindi kigumu chenye maumivu ya kimwili au kihisia?	0 1 2 3 4 5 6 7 8 9 10
SEASA5	How certain are you that you could control future suicidal thoughts if you lost an important relationship?<br>Kwa kiasi gani una uhakika kwamba utaweza kuyazuia mawazo ya kutaka kujiua ikiwa kama	0 1 2 3 4 5 6 7 8 9 10

	utapoteza mahusiano yaliyo muhimu sana kwako?	
SEASA6	How certain are you that you could control future suicidal thoughts if you lost a job, could not find employment, or suffered a financial crisis?<br>Kwa kiasi gani una uhakika kwamba utaweza kuyazuia mawazo ya kutaka kujiua ikiwa kama utapoteza kazi yako, utashindwa kupata ajira, au utakuwa na hali mbaya ya kifedha?	0 1 2 3 4 5 6 7 8 9 10

10. Hopelessness Scale

Description: This questionnaire contains 9 statements. Please indicate if each statement describes your attitude for the past week including today.

Maelezo: Dodoso hili linajumuisha kauli 9. Tafadhali onesha ikiwa kama kila kauli inaelezea mtazamo wako kwa kipindi cha wiki moja iliyopita ukijumuisha na leo.

		**True**<br>**Kweli**	**False**<br>**Sikweli**
BHS1	I might as well give up because there is nothing I can do about making things better for myself.<br>Ninaweza nikakata tamaa kwasababu hakuna kitu ambacho ninaweza kufanya ili kufanya Maisha yangu yawe mazuri.	1	0
BHS2*	In the future, I expect to succeed in what concerns me most.<br>Kwa siku zijazo, ninategemea nitafanikiwa kwenye mambo yangu ya msingi zaidi.	1	0
BHS3	All I can see ahead of me is unpleasantness.<br>Yote ninayoyatarajia kuja kwangu ninayaona kuwa si ya kufurahisha.	1	0
BHS4	I don't expect to get what I really want.<br>Sitarajii kupata vitu ninavyovihitaji zaidi	1	0
BHS5	Things just won't work out the way I want them to.<br>Mambo yangu hayaendi kama vile ninavyotaka mimi.	1	0
BHS6	I never get what I want, so it's foolish to want anything.<br>Sipati vile ninavyovitaka, kwahivyo ni ujinga kutaka chochote.	1	0

BHS7	It is very unlikely that I will get any real satisfaction in the future.<br>Hamna uhakika kabisa kama nitarizika kwa siku zijazo.	1	0
BHS8	The future seems vague and uncertain to me.<br>Naona kuwa hatima yangu haieleweki na haina uhakika.	1	0
BHS9	There's no use in really trying to get anything I want, because I probably won't get it.<br>Hakuna maana yoyote ya kujaribu kutafuta kitu chochote ninachohitaji, kwasababu inawezekana sitakipata.	1	0

11. Brief Reasons for Living Inventory (BRFL)

Description: Many people have thought about suicide at least once. The following are reasons people sometimes give for NOT committing suicide. We would like to know how important each of these possible reasons are to you at this time in your life as a reason to NOT kill yourself.

Maelezo: Watu wengi huwa na mawazo ya kujiua angalau mara moja. Zifuatazo ni sababu ambazo mara nyingine watu hutoa kwa KUTOJIUA. Tungependa kufahamu kiwango cha umuhimu wa kila sababu kwako katika kipindi hiki cha maisha yako, kama sababu ya KUTOJIUA.

		Not at all important<br>Sio muhimu kabisa	Quite unimportant<br>Sio muhimu	Somewhat unimportant<br>Sio muhimu kiasi	Somewhat important<br>Muhimu kiasi	Quite important<br>Muhimu	Extremely important<br>Muhimu kabisa
BRFL1	I am afraid of death<br>Naogopa kifo	1	2	3	4	5	6
BRFL2	My family depends upon me and needs me<br>Familia yangu inanitegemea na inanihitaji	1	2	3	4	5	6
BRFL3	I do not want to die<br>Sitaki kufa	1	2	3	4	5	6
BRFL4	The effect on my children could be harmful<br>Madhara kwa watoto wangu yangekua makubwa sana	1	2	3	4	5	6
BRFL5	I love and enjoy my family too much and could not leave them	1	2	3	4	5	6

	Naipenda na kuifurahia (kuienjoy) familia yangu sana na nisingeweza kuwaacha						
BRFL6	My religious beliefs forbid it<br>Imani yangu ya kidini inanikataza	1	2	3	4	5	6
BRFL7	I want to watch my children as they grow<br>Nataka niwaone watoto wangu wakikua	1	2	3	4	5	6
BRFL8	I am concerned about what others would think of me<br>Nina mashaka na jinsi watu wengine watakavyonifikiria	1	2	3	4	5	6
BRFL9	I consider it morally wrong<br>Ninaona kwamba hili ni kosa kimaadili	1	2	3	4	5	6
BRFL10	I am afraid of the actual "act" of killing myself (the pain, blood, violence)<br>Ninaogopa "tendo" lenyewe la kujiua (maumivu, damu, ukatili)	1	2	3	4	5	6
BRFL11	I would not want people to think I did not have control over my life<br>Nisingetaka watu wafikiri kwamba sikua na uwezo wa kuendesha maisha yangu	1	2	3	4	5	6
BRFL12	I believe I can find purpose in life, a reason to live<br>Ninaamini ninaweza kupata kusudi katika maisha, sababu ya kuishi	1	2	3	4	5	6

12. BSI Anxiety Subscale (BSI)

Description: I am going to read a list of problems people sometimes have. Please listen to each one carefully and tell me how much that problem has distressed or bothered you during the past 7 days, including today.

Maelezo: Nitasoma orodha ya matatizo ambayo watu huwa nayo. Tafadhali sikiliza kila moja kwa makini na kuniambia ni kiasi gani kila moja limekusikitisha au kukusumbua katika siku saba zilizopita ikiwa ni pamoja na leo.

		Not at all Hapana kabisa	A little bit Kiasi kidogo	Moderate Wastani	Quite a bit Kiasi kikubwa	Extremely Kiasi kikubwa sana
BSI1	Nervousness or shakiness inside.<br>Wasiwasi wa ndani	0	1	2	3	4
BSI2	Feeling tense or keyed up.<br>Kujisikia nafsi inahangaika/ kubanwa ndani	0	1	2	3	4
BSI3	Suddenly scared for no reason.<br>Unaogopa ghafla bila sababu	0	1	2	3	4
BSI4	Spells of terror or panic.<br>Vipindi vya hofu au mchecheto (kupanik)	0	1	2	3	4
BSI5	Feeling so restless you couldn't sit still.<br>Kujisikia kukosa utulivu	0	1	2	3	4
BSI6	Feeling fearful.<br>Hisia za woga na hofu	0	1	2	3	4

13. Beliefs about Medicines Questionnaire (BMQ)

Description: I'm going to read you some statements about feelings people may have about their HIV medications. Please listen to each statement and tell me how much you disagree or agree with each statement.

Maelezo: Nitakusomea kauli za hisia ambazo watu wanaweza kuwa nazo kuhusu dawa za VVU. Tafadhali sikiliza kila kauli kisha nieleze ni kwa kiasi gani hukubaliani au unakubaliana na kila kauli.

		**Strongly Disagree** Napinga kabisa	**Disagree** Napinga	**Uncertain** Sina hakika	**Agree** Nakubali	**Strongly Agree** Nakubali kabisa
BMQ1	My long-term health depends on my ARV medication. Afya yangu ya muda mrefu inategemea dawa za kurefusha maisha.	1	2	3	4	5
BMQ2*	Having to take ARVs for the rest of my life worries me. Kuhitaji kutumia dawa za kurefusha maisha kwa maisha yangu yote kunanitia hofu.	1	2	3	4	5
BMQ3	My future would be impossible without my ARVs. Maisha yangu yajayo yasingewezekana bila dawa zangu za kurefusha maisha.	1	2	3	4	5
BMQ4	Without my ARVs I would become very ill. Bila dawa zangu za kurefusha maisha ningekuwa mgonjwa sana.	1	2	3	4	5
BMQ5*	I sometimes worry about the long-term effects of my ARVs. Wakati mwingine ninaogopa kuhusu athari za muda mrefu za dawa zangu za kurefusha maisha.	1	2	3	4	5
BMQ6*	I don't understand my ARVs at all. Dawa zangu za kurefusha maisha ni fumbo kwangu.	1	2	3	4	5
BMQ7	My health in the future will depend on my ARVs. Afya yangu wakati ujao itategemea dawa zangu za kurefusha maisha.	1	2	3	4	5
BMQ8*	My ARVs disrupt my life. Dawa zangu za kurefusha maisha zinavuruga maisha yangu.	1	2	3	4	5
BMQ9*	I sometimes worry about becoming too dependent on my ARVs.	1	2	3	4	5

	Wakati mwingine ninakuwa na hofu kuhusu kuzitegemea dawa zangu za kurefusha maisha kupita kiasi.					
BMQ10	My ARVs protect me from my health becoming worse. Dawa zangu za kurefusha maisha zinanikinga afya yangu isiwe mbaya zaidi.	1	2	3	4	5

14. HIV Stigma Scale (HSS)

Description: This set of questions asks about some of your experiences, feelings, and opinions about how people with HIV feel and how they are treated.

Maelezo: Maswali haya yanaulizia kuhusu uzoefu, hisia, maoni yako juu ya jinsi ambavyo watu wanaoishi na VVU wanajisikia na wanavyotendewa.

		**Strongly Disagree** **Napinga kabisa**	**Disagree** **Napinga**	**Agree** **Nakubali**	**Strongly Agree** **Nakubali kabisa**
HSS1	I feel guilty because I have HIV. Ninajisikia mwenye hatia kwasababu nina VVU.	1	2	3	4
HSS2	People's attitudes about HIV make me feel worse about myself. Mtazamo wa watu kuhusu VVU unanifanya nijisikie vibaya juu ya nafsi yangu.	1	2	3	4
HSS3	Telling someone I have HIV is risky. Ni hatari kumwambia mtu kuwa nina VVU.	1	2	3	4
HSS4	I work hard to keep my HIV a secret. Ninafanya juhudi kubwa kufanya siri hali yangu ya VVU.	1	2	3	4
HSS5	I feel I'm not as good as others because I have HIV.	1	2	3	4

	Sijisikii kuwa mtu mzuri kama wengine kwasababu nina VVU.				
HSS6	People with HIV are treated like outcasts.<br>Watu wenye VVU wanachukuliwa/wanatendewa kama watu waliotengwa.	1	2	3	4
HSS7	Most people believe a person who has HIV is dirty.<br>Watu wengi wanaamini kuwa mtu mwenye VVU ni mchafu.	1	2	3	4
HSS8	I am very careful about who I tell I have HIV.<br>Nipo makini sana kuhusu mtu ninayemwambia kuwa nina VVU.	1	2	3	4
HSS9	Most people are uncomfortable around someone with HIV.<br>Watu wengi hawana raha (utulivu) wanapokuwa karibu na mtu mwenye VVU.	1	2	3	4

DISC6	In total, how many people outside of this clinic know your HIV status?<br>Kwa ujumla, ni watu wangapi nje ya kliniki hii ambao wanajua hali yako ya kuwa na VVU?	Enter number of people Andika namba ya watu:			
Ask Items HSS10-12 ONLY if the patient has disclosed their HIV to more than one person (DISC6 >1)					
		**Strongly Disagree Napinga kabisa**	**Disagree Napinga**	**Agree Nakubali**	**Strongly Agree Nakubali kabisa**
HSS10	People avoid touching me if they know I have HIV.<br>Watu wanaepuka kunigusa ikiwa wanajua kuwa nina VVU	1	2	3	4

HSS11	People I care about stopped calling after learning I have HIV.<br>Watu ninaowajali wameacha kunipigia simu baada ya kujua kuwa nina VVU	1	2	3	4
HSS12	I have lost relationships with friends or family members by telling them I have HIV.<br>Nimepoteza marafiki kwa kuwaambia kuwa nina VVU	1	2	3	4

15. HIV Acceptance: Adaptation of Illness Cognition Questionnaire (ICQ)

Description: You will hear a list of statements related to living with a positive HIV status. Please think about each statement and then select the answer which tell how often you feel this way.

Maelezo: Utasikia orodha ya kauli zinazohusiana na kuishi na VVU. Tafadhali fikiria kila kauli kisha uchague jibu linaloelezea ni mara ngapi unajisikia hivi.

		Not at all<br>Hapana hata kidogo	Somewhat<br>Kiasi fulani	To a large extent<br>Kiasi kikubwa	Completely<br>Kiasi kikubwa sana
ICQ1	I have learned to live with my HIV status.<br>Nimejifunza kuishi na hali yangu ya VVU.	1	2	3	4
ICQ2	I can accept my HIV status well.<br>Ninaweza kuikubali hali yangu ya VVU vizuri.	1	2	3	4
ICQ3	I can have a complete life with my HIV status.<br>Ninaweza kuishi kikamilifu na hali yangu ya VVU.	1	2	3	4
ICQ4	I can handle the problems related to my HIV status.<br>Ninaweza kumudu matatizo yanayohusiana na hali yangu ya VVU	1	2	3	4
ICQ5	I think I can handle the problems related to my HIV status, even if the HIV gets worse.<br>Nafikiri ninaweza kumudu matatizo yanayohusiana na hali	1	2	3	4

	yangu ya VVU, hata kama hali ya VVU itakuwa mbaya zaidi.				
ICQ6	I accept that there will be some life changes because of my HIV status. Nakubali kwamba kutakuwa na mabadiliko fulani katika maisha kwasababu ya hali yangu ya VVU	1	2	3	4
ICQ7	I believe that a person with HIV can live a long and healthy life. Ninaamini kwamba mtu aliye na VVU anaweza kuishi maisha marefu na yenye afya.	1	2	3	4

16. Perceived Availability of Social Support (PASS)

Description: The following questions have to do with the support you get from people in your life. I'm going to read you a series of questions about the different types of help people might give you. Please tell me whether someone would be available to provide that kind of help or support if you needed it. Remember that I'm not asking whether or not you need this kind of help at this time, but whether someone could help you if you needed it.

Maelezo: Maswali yafuatayo yanahusika na msaada unaopata toka kwa watu walioko maishani mwako. Nitakusomea maswali kadhaa kuhusu aina tofauti za msaada unaoweza kupewa na watu. Tafadhali niambie iwapo kuna mtu atapatikana kukupatia aina hiyo ya usaidizi ikiwa ungeuhitaji. Kumbuka kwamba sikuulizi iwapo unahitaji aina hii ya msaada kwa wakati huu au laa, bali ni iwapo mtu fulani angekusaidia kama ungeuhitaji.

		**Definitely not Hapana kabisa**	**Probably not Huenda Hapana**	**Possibly Inawezekana**	**Probably yes Huenda ndiyo**	**Definitely yes Ndiyo kabisa**
PASS1	Would someone be available to talk to you if you were upset, nervous, or depressed? Je, kuna mtu wa kuongea na wewe kama utakasirika, kuwa na wasiwasi, au kuwa na msongo wa mawazo?	1	2	3	4	5
PASS2	Is there someone you could contact if you wanted to talk about an important personal problem you were having? Je, kuna mtu unayeweza kuwasiliana naye kama	1	2	3	4	5

	ungetaka kuongea tatizo fulani muhimu na la binafsi ulilo nalo?					
PASS3	Is there someone who would help take care of you if you had to stay in bed for several weeks?<br>Je, kuna mtu ambaye angekutunza kama ungehitaji kukaa kitandani kwa wiki kadhaa?	1	2	3	4	5
PASS4	Is there someone you could turn to if you needed to borrow 2,000 TSh, needed to get a ride to the doctor or clinic, or needed some other small immediate help?<br>Je, kuna mtu ambaye ungemfuata kama ungehitaji kukopa shilingi 2000, ungehitaji kupata lifti kwenda kwa daktari au kliniki, au ungehitaji msaada mwingine mdogo wa haraka sana?	1	2	3	4	5
PASS5	Is there someone you could turn to if you needed to borrow some money to help pay your rent for one month?<br>Je, kuna mtu ambaye ungeweza kumfuata kama ungehitaji kukopa pesa kwaajili ya kulipia kodi ya nyumba kwa mwezi mmoja?	1	2	3	4	5
PASS6	Would the people in your personal life give you information, suggestions, or guidance if you needed it?<br>Je, watu walioko kwenye maisha yako binafsi wangekupa taarifa, mapendekezo, au maelekezo ikiwa ungehitaji?	1	2	3	4	5

PASS7	Is there someone you could turn to if you needed advice to help make a decision?<br>Je, kuna mtu ambaye unaweza kumwendea kama ungehitaji ushauri wa kukusaidia kufanya maamuzi?	1	2	3	4	5

17. History of Mental Health Treatment (HXMI)

HXMI1	Have you ever spoken to a counselor or mental health worker about emotional problems you were having?<br>Je, umeshawahi kuongea na mshauri au mhudumu wa afya ya akili kuhusu matatizo ya kihisia ulipokuwanayo?	0. No<br>Hapana<br>1. Yes<br>Ndiyo
HXMI1a	3-MONTH ONLY<br><br>(IF YES TO HXMI1): Have you spoken to a counselor or mental health worker about emotional problems you were having IN THE LAST 3 MONTHS (since you first enrolled in this study)?<br>Je, umewahi kuzungumza na mshauri au mhudumu wa afya ya akili kuhusu matatizo ya hisia ulipokuwa nayo KATIKA MIEZI 3 ILIYOPITA (tangu ulipojiandikisha kwenye utafiti huu)?	0. No<br>Hapana<br>1. Yes<br>Ndiyo<br>1.
HXMI1b	3-MONTH ONLY<br><br>IF YES TO HXMI1a): Did you speak to the counselor because you were referred or encouraged to do so by someone on this research team?<br>Je, ulizungumza na mshauri kwa sababu ulielekezwa au kuhimizwa kufanya hivyo na mtu kutoka kwenye timu hii ya utafiti?	0. No<br>Hapana<br>1. Yes<br>Ndiyo<br>2.
HXMI1c	3-MONTH ONLY<br><br>(IF YES TO HXMI1): How many times did you meet with a counselor or mental health worker in the LAST 3 MONTHS?	_________<br>3. *enter number / ingiza idadi*

	Ulikutana na mshauri au mhudumu wa afya ya akili mara ngapi KATIKA MIEZI 3 ILIYOPITA?	
HXMI1d	3-MONTH ONLY<br><br>(IF YES TO HXMI1): Did you start taking a new medication to help with mental health or emotional problems you were having IN THE LAST 3 MONTHS (Since you first enrolled in this study)?<br><br>Je, umeanza kumeza dawa mpya ili kukusaidia na matatizo ya afya ya akili au matatizo ya kihisia ulipokuwa nayo KATIKA MIEZI 3 ILIYOPITA (tangu ulipojiandikisha kwenye utafiti huu	0. No<br>Hapana<br>1. Yes<br>Ndiyo
HXMI2	BASELINE ONLY<br><br>Have you seen a traditional healer for support or treatment for emotional problems you were having?<br>Je, umekwenda kwa mganga wa kienyeii/jadi kwa ajili ya msaada/matibabu kuhusu matatizo ya kihisia ulipokuwanayo?	0. No<br>Hapana<br>1. Yes<br>Ndiyo
HXMI3	BASELINE ONLY<br><br>Have you seen a religious leader for support or treatment for emotional problems you were having?<br>Je, umekwenda kwa kiongozi wa kidini kwa ajili ya msaada au maombi/swala ya uponyaji kuhusu matatizo ya kihisia ulipokuwanayo?	0. No<br>Hapana<br>1. Yes<br>Ndiyo
HXMI4	Have you ever taken medication to help with mental health or emotional problems you were having?<br><br>Je, umewahi kumeza dawa ili kukusaidia na matatizo ya afya ya akili au matatizo ya kihisia ulipokuwanayo?	2. No<br>Hapana<br>3. Yes<br>Ndiyo<br><br>If yes, describe:<br><br>0. Kama ndiyo, elezea;

HXMI5	Have you ever been diagnosed with any mental illness?<br>Umewahi kugundulika na ugonjwa wowote wa akili?	1. No<br>Hapana<br>2. Yes<br>Ndiyo<br>If yes, name of mental illness:<br>Nini jina la huo ugonjwa wa akili?

18. Quality of Life (QOL)

Description: Please assess your feelings and give the answer that best describes how you are feeling today.

Maelezo: Tafadhali tathmini hisia zako na utoe jibu linalofafanua vizuri zaidi jinsi unavyojihisi leo.

		**Very Poor** **Mbaya sana**	**Poor** **Mbaya**	**Neither Poor Nor Good** **Si mbaya wala si nzuri**	**Good** **Nzuri**	**Very Good** **Nzuri sana**
QOL1	How would you rate your quality of life?<br>Je unaweza kusema hali ya ubora wa Maisha yako ikoje?	1	2	3	4	5
		**Very Dissatisfied** **Sijarizika kabisa**	**Dissatisfied** **Sijarizika**	**Neither Satisfied Nor Dissatisfied** **Haipo kwenye kurizika wala kutorizika**	**Satisfied** **Nimerizika**	**Very Satisfied** **Nimerizika kabisa**
QOL2	How satisfied are you with your health?<br>Je, kwa kiasi gani umeridhika na hali yako ya kiafya?	1	2	3	4	5

MOTIV SECTION ASKED AT BASELINE ONLY

19. Participant Motivation (MOTIV)

Description: We would like to understand how you feel about being in this study. Now you will hear a list of statements about your feelings about taking part in this study. Please choose how much you agree or disagree with each statement.

Maelezo: Tungependa kuelewa jinsi unavyojisikia kuhusu kuwa katika utafiti huu. Sasa utasikia orodha ya kauli kuhusu hisia zako kuhusu kushiriki katika utafiti huu. Tafadhali chagua ni kwa kiasi gani unakubali au hukubaliani na kila kauli.

		Strongly Disagree Sikubali kabisa	Disagree Sikubali	Agree Nakubali	Strongly Agree Nakubali kabisa
MOTIV1	I am enthusiastic about taking part in this research study.<br>Nina shauku ya kushiriki katika utafiti huu.	1	2	3	4
MOTIV2	I feel motivated to complete the activities in this research study.<br>Ninahisi kuhamasika kukamilisha shughuli zote katika utafiti huu.	1	2	3	4
MOTIV3	I feel confident that I will complete all of the activities involved in this research study.<br>Nina uhakika kwamba nitakamilisha shughuli zote zinazohusika katika utafiti huu.	1	2	3	4
MOTIV4	I am enthusiastic about participating in counseling.<br>Nina shauku ya kushiriki katika ushauri nasaha.	1	2	3	4
MOTIV5	I am motivated to participate in counseling.<br>Nimehamasishwa kushiriki katika ushauri nasaha.	1	2	3	4
MOTIV6	I feel confident that I will actively participate in the counseling sessions.<br>Ninajiamini kuwa nitashiriki kikamilifu katika vikao vya ushauri.	1	2	3	4

THE REMAINING SECTIONS ARE ASKED AT 3-MONTHS ONLY ONLY

20. Participant Feedback Overall Study (STUDY)

Description: We would like to understand how you felt being in this study. Now you will hear a list of statements about what it may have been like to take part in this study. Please choose how much you agree or disagree with each statement.

Maelezo: Tungependa kufahamu ulijisikiaje kushiriki katika utafiti huu. Sasa utasikia orodha ya kauli kuhusu jinsi ilivyokuwa kushiriki katika utafiti huu. Tafadhali chagua ni kwa kiasi gani unakubaliana au kutokukubaliana na kila kauli.

		Strongly Disagree Sikubali kabisa	Disagree Sikubali	Agree Nakubali	Strongly Agree Nakubali kabisa
STUDY1	My experience with the study was positive. Uzoefu wangu katika kushiriki kwenye utafiti huu ulikua mzuri.	1	2	3	4
STUDY2	If I had the chance I would take part in this study again. Iwapo ningepata fursa ya kushiriki tena kwenye utafiti huu, ningeshiriki kwa awamu nyingine.	1	2	3	4
STUDY3	I would recommend this study to other people attending the clinic. Ningeupendekeza utafiti huu kwa watu wengine wanaofika kliniki.	1	2	3	4
STUDY4	I trust the research staff to keep my information confidential. Ninaamini kuwa watafiti watatunza taarifa zangu kwa usiri.	1	2	3	4
STUDY5	I felt comfortable answering the survey questions. Nilijisikia kujibu maswali ya dodoso.	1	2	3	4
STUDY6	The questions I was asked in the surveys were easy for me to understand. Maswali niliyoulizwa kwenye dodoso yalikua rahisi kwangu kuelewa.	1	2	3	4

21. Participant Intervention Feedback (INT)

Description: You will now hear a list of statements about what it has been like to talk with the counselors in the study. Please choose how much you agree or disagree with each statement.
Maelezo: Sasa utasikia orodha ya kauli kuhusu ni jinsi gani ilivyokuwa kuongea na mshauri kwenye utafiti. Tafadhali chagua ni kwa kiasi gani unakubaliana au kutokukubaliana na kila kauli.

		Strongly Disagree Sikubali kabisa	Disagree Sikubali	Agree Nakubali	Strongly Agree Nakubali kabisa
INT1	I liked the counselor who I met with. Nilimpenda mshauri niliyekutana nae.	1	2	3	4
INT2	I felt that the counselor listened to me and responded to my questions and concerns. Nilijisikia kua mshauri alinisikiliza na alijibu vizuri maswali yangu.	1	2	3	4
INT3	The content of the counseling was useful to me. Maudhui ya kipindi cha ushauri yalikua na manufaa kwangu.	1	2	3	4
INT4	The counseling helped me to manage and reduce thoughts of suicide. Maudhui ya ushauri yalinisaidia kuyamudu na kupunguza mawazo ya kujiua?	1	2	3	4
INT5	The counseling made me more motivated to attend the HIV clinic. Ushauri umenifanya nihamasike zaidi kuhudhuria kliniki ya VVU.	1	2	3	4
INT6	The counseling made me more motivated to take my HIV medication. Ushauri umenifanya nihamasike zaidi kumeza dawa zangu za kupunguza makali ya VVU.	1	2	3	4
INT7	The counseling helped to educate me about HIV and the importance of HIV treatment. Ushauri ulinisaidia kunielimisha kuhusu VVU na umuhimu wa tiba ya VVU	1	2	3	4

INT8	The counseling helped me to disclose my HIV status to others.<br>Ushauri umenisaidia kuwashirikisha wengine kuhusu hali yangu ya maambukizi ya VVU.	1	2	3	4
INT9	The counseling helped me to feel more hopeful about my future.<br>Ushauri umenisaidia kuwa na matumaini zaidi kuhusu wakati ujao.	1	2	3	4
INT10	The counseling helped me to cope with HIV stigma.<br>Ushauri umenisaidia kukabiliana na unyanyapaa ya VVU.	1	2	3	4

		**Too Little Mchache sana**	**Too Much Mwingi sana**	**The Right Amount Wa kawaida**
INT11	Think about the time you spent meeting with the counselor. Do you think that this took too little time, too much time, or just the right time?<br>Fikiria kuhusu muda uliotumia kukutana na mshauri. Je, unafikiri hii ilichukua muda mchache sana, mwingi sana au muda wa kawaida?	1	2	3
		**Too Little Mara chache sana**	**Too Much Mara nyingi sana**	**The Right Amount Idadi ya kawaida**
INT12	Think about the number of times you met with a counselor during this study. Do you think it was too little times, too many times, or just the right number of times?<br>Fikiria kuhusu idadi ya vipindi ulivyookutana na mshauri wakati wa utafiti huu. Je, unafikiri ilikua mara chache sana, mara nyingi sana au idadi ya kawaida?	1	2	3

Description. How useful did you find the following activities?
Maelezo: Je, shughuli zifuatazo zilikua na manufaa kiasi gani kwako?

		Not very useful<br>Hakikuwa na manufaa sana	A little useful<br>Manufaa kidogo	Very useful<br>Manufaa sana
INT13	**INTERVENTION ONLY:**<br>How useful did you find Session 1? As you may recall, in the first session we talked about the concept of recovery. When we said, 'Every day, people find a way to recover from feelings of sadness and they gain the ability to live a life of hope and purpose,' we also talked about the four pillars, values, and problem-solving and finally, we discussed the safety plan<br>Kwa kiasi gani kipindi cha kwanza kilikuwa na manufaa? Kama unavyoweza kukumbuka, katika kikao cha kwanza tulizungumza juu ya dhana ya kupona. Tuliposema, ‘Kila siku, watu hutafuta njia ya kupata nafuu kutokana na hisia za huzuni na kupata uwezo wa kuishi maisha yenye tumaini na kusudi,’ tulizungumza pia kuhusu nguzo nne, maadili, na kutatua matatizo na mwisho tulijadili kuhusu mpango wa usalama.	1	2	3
INT14	**INTERVENTION ONLY:**<br>How useful did you find Session 2? As you may remember, in the second session we talked about Stigma Reduction Through Storytelling and Problem Solving, and I started by giving you the story of a young girl. Do you remember?<br>Kwa kiasi gani kipindi cha pili kilikuwa na manufaa? Kama unavyoweza kukumbuka, katika kikao cha pili tulizungumza juu ya Kupunguza Unyanyapaa Kupitia Hadithi na Utatuzi wa Matatizo, na nilianza kwa kukupa ile hadithi ya yule dada. Je unakumbuka?	1	2	3

INT15	**INTERVENTION ONLY:**<br>How useful did you find Session 3? As you may remember, in the third session we talked about the action plan.<br>Kwa kiasi gani kipindi cha tatu kilikuwa na manufaa? Kama unavyoweza kukumbuka, katika kipindi cha tatu tulizungumza kuhusu mpango kazi.	1	2	3

Description. How useful did you find the following activities?
Maelezo: Je, shughuli zifuatazo zilikua na manufaa kiasi gani kwako?

		Not very useful<br>Hakikuwa na manufaa sana	A little useful<br>Manufaa kidogo	Very useful<br>Manufaa sana
INT16	**COMPARISON ONLY:**<br>How useful did you find the Safety Planning session?<br>Kwa kiasi gani kipindi cha mpango wa usalama kimekua na manufaa kwako?	1	2	3

Description. How useful did you find the following activities?
Maelezo: Je, shughuli zifuatazo zilikua na manufaa kiasi gani kwako?

		Not very useful<br>Haukuwa na manufaa sana	A little useful<br>Manufaa kidogo	Very useful<br>Manufaa sana
INT17	**ALL PARTICIPANTS:**<br>How useful did you find the text message check-ins?<br>Kwa kiasi gani umeona umuhimu wa kupokea ujumbe wa maandishi wa kukujulia hali?	1	2	3

INT18	**ALL PARTICIPANTS:**<br>Did you call the counselor for any booster sessions?<br>Je, ulimpigia simu mshauri kwa vipindi vyovyote vya nyongeza?	4. No<br>Hapana<br>2. Yes. If yes, how many sessions?<br>Ndiyo ______________

		Not very useful Havikuwa na manufaa sana	A little useful Manufaa kidogo	Very useful Manufaa sana
INT19	**IF THEY ATTENDED BOOSTER SESSIONS:** How useful did you find the booster sessions? Kwa kiasi gani vipindi vya nyongeza vimekua na manufaa kwako?	1	2	3

22. Other Study Participation

OTHER1	During your participation in this study, were you enrolled in any other research study with KCMC?<br>Wakati wa ushiriki wako kwenye utafiti huu, je ulikuwa unashiriki kwenye utafiti mwingine wowote unaoratibishwa na watu wa kutoka KCMC?	0. No Hapana<br>1. Yes Ndiyo<br>If yes, describe. Include whether the study included mental health counseling or support for HIV care engagement:<br>Kama ndiyo, elezea. Jumuisha kama utafiti ulihusisha ushauri wa afya ya akili au msaada wa kushiriki kwenye huduma ya VVU:

23. Open-Ended Feedback

**FB1**	Tell me how it was talking to the counselor. What was most helpful about talking to a counselor?

	Nieleze ilikuwaje kuzungumza na mshauri. Ni nini kilikua cha msaada zaidi kuhusu kuzungumza na mshauri?
**FB2**	What was least helpful about talking to a counselor? What do we need to improve?<br>Ni kitu gani kimekua cha msaada ulipozungumza na mshauri? Kitu gani tunapaswa kuboresha?
**FB3**	Did talking to the counselor have any impact the way you feel about your HIV status? How did it influence you? (probe: acceptance, hopefulness, stigma)<br>Je, kuzungumza na mshauri kumeathiri vipi jinsi unavyojisikia kuhusu hali yako ya VVU? Je, ilikuathiri vipi? (dodosa: kukubalika, matumaini, unyanyapaa)
**FB4**	Did talking to the counselor impact your suicidal thoughts or other aspects of your mental health? How did it influence you? (probe: hope, coping strategies, social support)<br>Je, kuzungumza na mshauri kumegusa mawazo yako ya kujiua au vipengele vingine vya afya yako ya akili? Je, imekugusa kwa namna gani? (chunguza: tumaini, mikakati ya kukabiliana, usaidizi wa kijamii)

**FB5**	Did your participation in counseling impact your motivation to take part in HIV care? How did it influence you?<br>Je, ushiriki wako katika unasihi uliathiri/uliamsha hali yako ya kushiriki katika huduma za VVU? Je, ilikuathiri/iliamsha vipi?
**FB6**	Is there anything else you would like to say about your experience, or any questions before we leave?<br>Je, kuna kitu kingine chochote ambacho ungependa kusema kuhusu uzoefu wako au swali lolote kabla hatujamaliza?

Thank you so much for your feedback and insight in completing this survey. We appreciate your input and will hope to use this information to improve care for people living with HIV in Moshi. Do you have any questions?
Asante sana kwa mrejesho na uelewa wako katika kukamilisha dodoso hili. Tunathamini sana mchango wako na tunatumaini kutumia taarifa hizi kuboresha huduma ya watu wanaoishi na VVU mjini Moshi. Je una swali lolote.

TO BE COMPLETED BY RESEARCH ASSISTANT

TIME	Elapsed time for survey (in minutes):

BASELINE SURVEY: COMPLETE RANDOMIZATION

****INSTRUCTIONS TO RA:** At this point, explain to the participant that you will now select the envelope to learn what type of counseling they will receive.

Enter the result in REDCap and complete the survey before connecting them to the telehealth counselor.

RANDOMIZATION	Enter randomization result	**1. Comparison (Safety Planning)**<br><br>**2. Intervention (IDEAS for Hope Intervention)**

AUTOMATICALLY RECORDED

TIME	Elapsed time for survey (in minutes):
