# Supplementary material for: Protocol for a pilot randomized controlled trial of a telehealth-delivered counseling intervention to reduce suicidality and improve HIV care engagement in Tanzania
Source: PLoS One. 2023 Jul 27;18(7):e0289119. doi: 10.1371/journal.pone.0289119 (PMC10374000; doi:10.1371/journal.pone.0289119)
Supplement: S4 Appendix — (PDF) [file pone.0289119.s006.pdf]

Study ID: __ - __ __ __
Date:

Clinical Trial Pre-Session Assessment

Description: I am going to ask you some questions about how you are feeling right now. Please answer these questions openly and honestly.

Maelezo: Nitakuuliza baadhi ya maswali kuhusu unavyojisikia sasa hivi. Tafadhali jibu maswali haya kwa uwazi na uaminifu.

		**Not at All** **Hapana kabisa**	**A Little Bit** **Kiasi kidogo**	**Somewhat** **Kiasi fulani**	**Very Much** **Kiasi kikubwa**
PRPO1	I feel down, depressed, or hopeless<br>Ninajihisi kuwa na huzuni, kusononeka au kukosa tumaini	0	1	2	3
PRPO2	I feel motivated to improve my situation<br>Nahisi kuhamasika kuboresha hali yangu	0	1	2	3
PRPO3	I feel hopeful about the future<br>Nina matumaini na siku zijazo	0	1	2	3
PRPO4	I feel capable and empowered to improve my situation<br>Nahisi kuwezeshwa kuboresha hali yangu	0	1	2	3

Description: Please indicate the number (0–10) that best describes how much distress you are experiencing right now.
Maelezo: Tafadhali taja namba (0-10) inayoashiria kwa karibu zaidi kiasi cha msongo unaopata sasa hivi

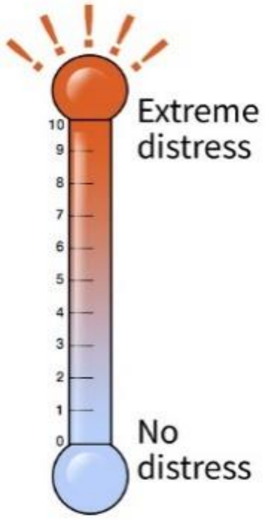

	Current Feelings	
	YES	NO
**1) Are you having any actual thoughts of killing yourself?**<br>*Hivi sasa unapata mawazo yoyote ya kujiua?*		
**2) Are you thinking about how you might do this?**<br>*Je, unawaza kuwa utalifanyaje hili jambo?*		
**3) Do you have some intention of acting on these thoughts?**<br>Mark no for "I have the thoughts but I will not do anything about them."<br>*Je, una nia yoyote ya kuyafanyia kazi hayo mawazo?*		

Description: I am going to ask you some questions about the HIV antiretroviral medications that you were prescribed to treat your HIV.

Maelezo: Nitakuuliza maswali kuhusu dawa za kurefusha maisha ambazo uliandikiwa kutibu VVU yako.

TAA3	Within the last 14 days, have you taken ARVs?<br>Katika kipindi cha siku 14 aumiezi 3 iliyopita, umewahi kumeza dawa za kupunguza makali ya VVU?	0. No<br>1. Yes
TAA4	Think about the last 14 days. On how many *days* did you miss taking your HIV pill(s)?<br>Katika siku 14 zilizopita ni *siku* ngapi ulikosa kumeza vidonge vyako vya VVU?	[***Write down days: 0-14***]

Clinical Trial Post-Session Assessment

Description: I am going to ask you some questions about how you are feeling now, after today's session. Please answer these questions openly and honestly.

Maelezo: Nitakuuliza baadhi ya maswali kuhusu unavyojisikia sasa, baada ya kipindi cha leo. Tafadhali jibu maswali haya kwa uwazi na uaminifu.

		**Not at All** **Hapana kabisa**	**A Little Bit** **Kiasi kidogo**	**Somewhat** **Kiasi fulani**	**Very Much** **Kiasi kikubwa**
PRPO1	I feel down, depressed, or hopeless<br>Ninajihisi kuwa na huzuni, kusononeka au kukosa tumaini	0	1	2	3
PRPO2	I feel motivated to improve my situation<br>Nahisi kuhamasika kuboresha hali yangu	0	1	2	3
PRPO3	I feel hopeful about the future<br>Nina matumaini na siku zijazo	0	1	2	3
PRPO4	I feel capable and empowered to improve my situation<br>Nahisi kuwezeshwa kuboresha hali yangu	0	1	2	3

Description: Please indicate the number (0–10) that best describes how much distress you are experiencing right now.
Maelezo: Tafadhali taja namba (0-10) inayoashiria kwa karibu zaidi kiasi cha msongo unaopata sasa hivi

PRPO5

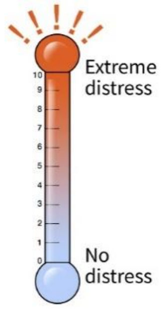

	Current Feelings	
	YES	NO
**PRPO6) Are you having any actual thoughts of killing yourself?**<br>*Hivi sasa unapata mawazo yoyote ya kujiua?*		
**PRPO7) Are you thinking about how you might do this?**<br>*Je, unawaza kuwa utalifanyaje hili jambo?*		
**PRPO8) Do you have some intention of acting on these thoughts?**<br>Mark no for "I have the thoughts but I will not do anything about them."<br>*Je, una nia yoyote ya kuyafanyia kazi hayo mawazo?*		
**PRPO9) If yes to number 3, what did you (the counselor) do to connect the participant with additional support?**		

Response Protocol to C-SSRS Screening

If participant responds "Yes" to Question 3 after the counseling session – you should inform the RA that a direct referral for mental health services is needed.

Depression Item Sources: PHQ-2, Hospital Anxiety and Depression Scale (HADS), Zung Self-Rating Depression Scale (SDS)
