# Supplementary material for: Protocol for a pilot randomized controlled trial of a telehealth-delivered counseling intervention to reduce suicidality and improve HIV care engagement in Tanzania
Source: PLoS One. 2023 Jul 27;18(7):e0289119. doi: 10.1371/journal.pone.0289119 (PMC10374000; doi:10.1371/journal.pone.0289119)
Supplement: S5 Appendix — (PDF) [file pone.0289119.s007.pdf]

Participant Study ID:

Counselor Initials:

Counseling Notes

Date of Contact:

Type of Contact:

<u>Data</u>

What happened in the session, what did the client say, what were your impressions of the client?

<u>Action</u>

How did you respond to the client, what counseling was provided, what goals were set?

<u>Plan</u>

What will the client do after the session, what will you do?
